# An assessment of prognostic immunity markers in breast cancer

**DOI:** 10.1038/s41523-018-0088-0

**Published:** 2018-10-29

**Authors:** Benlong Yang, Jeff Chou, Yaozhong Tao, Dengbin Wu, Xinhong Wu, Xueqing Li, Yan Li, Yiwei Chu, Feng Tang, Yanxia Shi, Linlin Ma, Tong Zhou, William Kaufmann, Lisa A Carey, Jiong Wu, Zhiyuan Hu

**Affiliations:** 1 Department of Breast Surgery, Shanghai Cancer Center, Shanghai, China; 2 Department of Oncology, Shanghai Medical College, Fudan University, Shanghai, China; 3 Collaborative Innovation Center for Cancer Medicine, Shanghai, China; 4 Department of Biostatistics, Wake Forest Baptist Medical Center, Winston-Salem, NC USA; 5 Lineberger Comprehensive Cancer Center, University of North Carolina at Chapel Hill, Chapel Hill, NC USA; 6 Department of Oncology, An-Steel Group Hospital, Anshan, Liaoning China; 7 Department of Breast Surgery, Hubei Cancer Hospital, Huazhong University of Science and Technology, Wuhan, Hubei China; 8 Department of Thyroid and Breast Surgery at the Fifth People’s Hospital, Fudan University, Shanghai, China; 9 Department of Immunology, Fudan University, Shanghai, China; 10 Department of Pathology, Huashan Hospital, Fudan University, Shanghai, China; 11 Department of Medicine, Sun Yat-Sen University Cancer Center, Guangzhou, China; 12 Shanghai Precision Diagnostics Co. Ltd., Shanghai, China; 13 Asystbio Laboratories LLC, Chapel Hill, NC USA; 14 North Carolina Cancer Hospital, Chapel Hill, NC USA; 15 Division of Hematology-Oncology UNC School of Medicine, University of North Carolina at Chapel Hill, Chapel Hill, NC USA

## Abstract

Tumor-infiltrating lymphocytes (TIL) and immunity gene signatures have been reported to be significantly prognostic in breast cancer but have not yet been applied for calculation of risk of recurrence in clinical assays. A compact set of 17 immunity genes was derived herein from an Affymetrix-derived gene expression dataset including 1951 patients (AFFY1951). The 17 immunity genes demonstrated significant prognostic stratification of estrogen receptor (ER)-negative breast cancer patients with high proliferation gene expression. Further analysis of blood and breast cancer single-cell RNA-seq datasets revealed that the 17 immunity genes were derived from TIL that were inactive in the blood and became active in tumor tissue. Expression of the 17 immunity genes was significantly (*p* < 2.2E-16, *n* = 91) correlated with TILs percentage on H&E in triple negative breast cancer. To demonstrate the impact of tumor immunity genes on prognosis, we built a Cox model to incorporate breast cancer subtypes, proliferation score and immunity score (72 gene panel) with significant prediction of outcomes (*p* < 0.0001, *n* = 1951). The 72 gene panel and its risk evaluation model were validated in two other published gene expression datasets including Illumina beads array data METABRIC (*p* < 0.0001, *n* = 1997) and whole transcriptomic mRNA-seq data TCGA (*p* = 0.00019, *n* = 996) and in our own targeted RNA-seq data TARGETSEQ (*p* < 0.0001, *n* = 303). Further examination of the 72 gene panel in single cell RNA-seq of tumors demonstrated tumor heterogeneity with more than two subtypes observed in each tumor. In conclusion, immunity gene expression was an important parameter for prognosis and should be incorporated into current multi-gene assays to improve assessment of risk of distant metastasis in breast cancer.

## Genomic testing: Tumor-infiltrating immune cells express detectable gene signature

The elevated expression of 17 immunity-related genes is associated with better outcomes among women with aggressive forms of estrogen receptor–negative breast cancer. Zhiyuan Hu from the University of North Carolina at Chapel Hill, USA, and colleagues identified the 17-gene set by analyzing a larger expression dataset from close to 2,000 patients. Single-cell sequencing revealed that the genes were turned on in a group of cancer-fighting immune cells known as tumor-infiltrating lymphocytes, but were inactive in circulating blood cells. The researchers incorporated the immunity-related genes into a larger panel of genes involved in proliferation, invasion and other relevant biological processes. The resulting 72-gene test was an accurate predictor of the risk for developing distant metastases. The findings suggest that immunity-related genes should be incorporated into current multi-gene prognostic assays for women with breast cancer.

## Introduction

Metastasis is the main cause of mortality for breast cancer patients. Factors such as cell cycle deregulation, stromal microenvironment, proteases, endothelial cells, myoepithelial cells and immunity status within a tumor can drive metastasis.^1^ Targeted inhibition of immune checkpoint function by antibodies against PD-1,^2,3^ PD-L1^4,5^ and CTLA4^6^ has revealed active anti-tumor, T cell-mediated immunity. Tumor-infiltrating lymphocytes (TIL) have been well-reported to play critical roles in response to chemotherapy and prognosis in breast cancer, specifically in triple-negative and HER2-positive breast cancers, with a survival benefit being seen in patients having >50% lymphocyte-predominant tumors.^7–9^ Immunity-related gene classifiers have also been reported to stratify prognosis in immune-benefit-enabled tumors comprised mostly of Basal-like, HER2-enhanced (HER2E), and Luminal B tumors.^10^ Quantitative assessment of anti-tumor immunity and responsiveness to immunotherapy represents an important new avenue of breast cancer research.

Gene expression profiles of primary tumors are highly predictive of distant metastasis^11–15^ in breast cancer and the genomic portrait is maintained between the primary tumor and its metastases.^1,16,17^ As the genetic and epigenetic properties of a primary tumor define its fate and capability to develop metastasis, the expression signatures of the primary tumor are prognostic and predict a patient’s outcome. Three multi-gene expression assays, PCR-based Oncotype DX (Genomic Health Inc., Redwood City, CA, USA),^15,18^ microarray-based MammaPrint (Agendia Inc., Amsterdam, Netherlands),^13,19^ and nanostring-based PAM50 Prosigna Assay (NanoString Technologies Inc., Seattle, WA, USA),^20–23^ have been widely used in clinical practice to determine the risk of recurrence in patients with breast cancer. Genes monitored in these assays mainly include drivers of cell proliferation, hormone receptors, HER2 and basal cytokeratins. The PAM50 expression assay with integration of breast cancer subtype and proliferation score in risk assessment was shown to provide better prognostic information in ER-positive, node-negative patients than Oncotype DX.^24^ Agreement between risk classifications based on Oncotype DX and PAM50 was as low as 54%, demonstrating substantial differences between the molecular classifiers in patient risk stratification.^25^ However, none of the current multi-gene expression assays have included the very important and prognosis-related immunity genes. To improve accuracy in evaluating risk of distant metastasis of breast cancer we created a new model that added immunity genes based on gene expression profiling.

## Results

### Prognostic immunity and proliferation genes in breast cancer

We analyzed 1951 Affymetrics gene expression profiles (AFFY1951) from 14 breast cancer cohorts with median follow-up of 7.12 years, median time-to-distant metastasis (DM) of 2.7 years and 481 DM events. There were 20% ER-negative, 69% ER-positive, and 11% unknown ER status in the AFFY1951 training data set. HER2 status for the 14 published cohorts was not provided (Supplementary Table 1). Two highly significant biological categories, immune response (*p* < 0.001) and cell cycle (*p* < 0.001) were identified with 119 and 71 genes in each category respectively (Supplementary Table 2). The Cox coefficient and the magnitude of change associated with distant metastasis-free survival (DMFS) were used for selection of immunity and cell cycle genes for further analysis. The top-ranked 17 immunity genes and 19 cell cycle genes were used for calculation of immunity and proliferation scores.

**Fig. 1:**
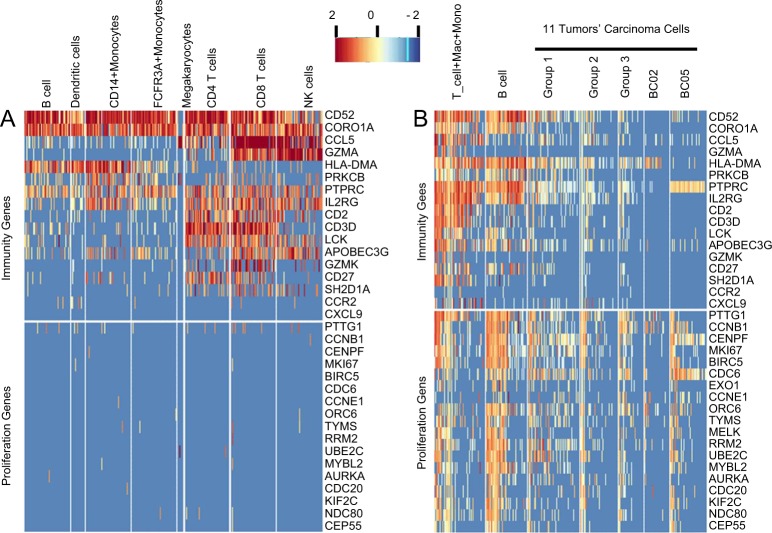
RNA-seq gene expression of 17 immunity genes and 19 proliferation genes in published PBMC single-cell dataset (Macosko et al. Cell 2015)^26^ and breast cancer solid tumor single cells (Chung et al. Nature Communications 2017).^27^ (A) Expression of immunity and proliferation genes in different PBMC cell types including B cells, CD4 T cells, CD8 T cells, NK cells, Monocytes and dendritic cells. (B) Expression of immunity and proliferation genes of single cells including breast tumors’ immune single cells labeled as T_cell + Mac + M & B_cell (Mac = macrophages, M = monocytes) and carcinoma single cells groups 1 to 3 (mixed carcinoma single cells from different tumors), BC02 and BC05 (carcinoma single cells from each individual tumor)

Single cell RNA-seq analysis of peripheral blood mononuclear cells (PBMC) using a publicly available dataset^26^ indicated that 15 of the 17 immunity genes, excepting CCR2 and CXCL9, were expressed in at least one of eight different types of immune cells. However, none of the 19 cell cycle genes were expressed, indicating that the immune cells are not proliferating in the blood (Fig. 1a). We further analyzed gene expression in single cells including both immune cells and tumor cells isolated from solid breast tumor tissues.^27^ The results showed that expression of the 17 immunity genes was evident in the two immune cell groups but sporadically or not in the five tumor carcinoma cell groups (Fig. 1b). A portion of the total immune cells in the 11 tumors (about 20% T cell group and 60% B cells) highly expressed the 19 proliferation genes while less than 20%, on average, of the carcinoma cells expressed the proliferation genes in this single cell RNA-seq data^27^ (Fig. 1b).

We next compared the 17 immunity genes with other immunity signature modules. The 17 immunity genes were representatives of 119 immunity genes with high correlation (Pearson’s correlation = 0.87, 95%CI: 0.86-0.88, *p* < 0.0001, *n* = 1951) derived by EPIG in the AFFY1951 dataset (Supplementary Figure 1A). We compared the 17-gene immunity signature with 500 other published immunity signatures (data not shown). The most highly correlated published immunity signatures were shown, as an example, in the TCGA breast cancer RNA-seq dataset (Supplementary Figure 1B). Correlation analysis revealed CD4 and CD8 T cell signatures, B cell signature, LCK signature, NK cell signature, Miller’s immune signature, the UNC immune signature, and Cluadin-low upregulated gene signature were all significantly positively correlated with the 17-gene immunity signature with correlations from 0.77 to 0.94, but negatively (lowest −0.51) correlated with the Claudin-low down-regulated gene signature (Supplementary Figure 1B). We also noticed that the 17-gene immunity signature was the most compact significant gene signature and therefore suitable for further analysis for clinical application.

**Fig. 2:**
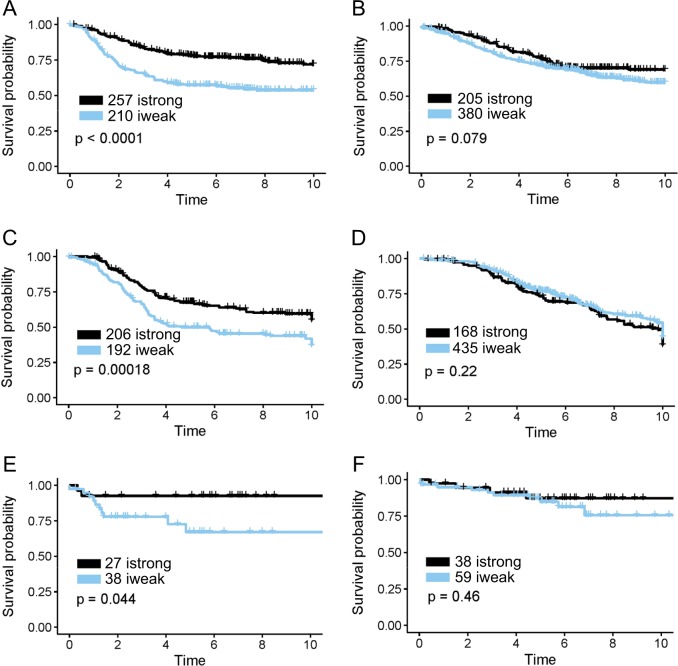
Survival plots of Immunity Score in different patient groups identified by proliferation and ER status in the AFFY1951 training dataset, two test datasets METABRIC and TARGETSEQ. Immunity Score demonstrated strongest outcome prediction in patients who were ER-negative and proliferation high in AFFY1951 (A) (*p* < 0.0001, *n* = 467), METABRIC (C) (*p* = 0.00018, *n* = 398) and TARGETSEQ (E) (*p* = 0.044, *n* = 65), but was insignificant in ER-positive and proliferation high patients in AFFY1951 (B) (*p* = 0.079, *n* = 585), METABRIC (D) (*p* = 0.22, *n* = 603) and TARGETSEQ (F) (*p* = 0.46, *n* = 97). High proliferation groups had proliferation scores no less than 50 and low proliferation groups had proliferation scores less than 50. Survival analysis of ER-negative or ER-positive and low proliferation patients were demonstrated in Supplementary Figure 1

Patients were first divided into two groups based on either immunity scores or proliferation scores. In general, patients who were in the immunity-strong group (istrong) had better prognosis (DMFS) than those in the immunity-weak group (iweak), and patients who were in the high proliferation group had worse prognosis than those in the low proliferation group in AFFY1951 (Supplementary Figure 2). However, further analysis of the training dataset AFFY1951 showed that immunity score was prognostic of DMFS only in patients who were proliferation-high and ER-negative, and patients in the istrong group displayed a significantly better outcome (Fig. 2a, *p* < 0.0001). Immunity score had no significant effect on DMFS in all other patients who were either proliferation-high & ER-positive (Fig. 2b) or proliferation-low regardless of ER status (Supplementary Figure 3A and B). The same results were replicated in two independent validation datasets, the publicly available gene expression dataset “METABRIC”^28^ of fresh-frozen breast tissues and our targeted RNA-seq dataset “TARGETSEQ” of breast cancer FFPE tissues, in proliferation-high and ER-negative (Fig. 2c,e) or ER-positive groups (Fig. 2d,f), and proliferation-low groups (Supplementary Figure 3C, D and E). Proliferation gene expression and ER status demonstrated significant impact on the prognostic value of immunity genes in breast cancer.

### The “Immunity-enhanced” group and immunity score in evaluation of risk of distant metastasis

**Fig. 3:**
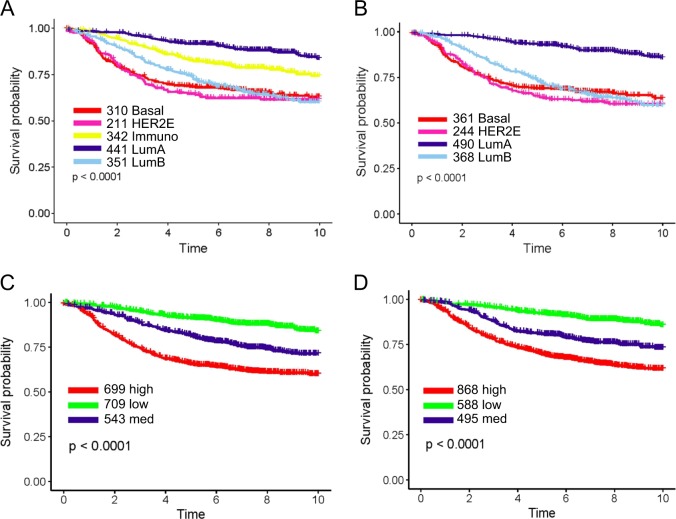
Comparison of survival analysis of iRDM and PAM50 in AFFY1951 breast cancer training dataset. Subtypes and risk groups are color-coded: Basal-like (Red), HER2E (Hot Pink), Immuno (Yellow), Luminal A (Dark Blue), Luminal B (Sky Blue), Normal-like (Green); low (Green), med (Dark Blue), and high (Red) risks. Kaplan-Meier plots were used to show Distant Metastasis-Free Survival (DMFS) by subtypes for iRDM (A) (*p* < 0.0001, *n* = 1655) and PAM50 (B) (*p* < 0.0001, *n* = 1463) and risk groups for iRDM (C) (*p* < 0.0001, *n* = 1951) and PAM50 (D) (*p* < 0.0001, *n* = 1951)

To further evaluate the significance of immunity genes and proliferation genes in prognosis, a 72-gene test panel, including the 17 immunity genes, 19 proliferation genes, 11 Basal genes, 14 ER genes, 3 HER2 genes, 2 invasion genes, and 6 housekeeper genes (Supplementary Table 3) was applied for subtype and immunity-adjusted risk of distant metastasis (iRDM) analysis. The five PAM50 breast cancer subtypes Luminal A (LumA), Luminal B (LumB), Basal-like (Basal), HER2-Enriched (HER2E) and Normal-like (Normal)^16,29,30^ were recaptured by the iRDM analysis. Interestingly, an additional group termed “Immunity-enhanced” (Immuno) was identified (Supplementary Figure 4A. The new group accounting for about 18% of tumors (Table 1) demonstrated high expression of the 17 immunity genes and low or sporadic expression of the other breast cancer biomarker genes. Comparison of heatmaps sorted by expression of immunity genes in both iRDM and PAM50 subtypes (Supplementary Figure 4A and B) using the AFFY1951 dataset showed that high immunity gene expression was present within each molecular subtype but not well-correlated with any of the other molecular markers. Excluding Normal-like and Mixed samples, iRDM subtypes demonstrated significant outcome prediction in the AFFY1951 dataset (Fig. 3a), very similar to the result of the PAM50 analysis (Fig. 3b) except for an additional Immunity-enhanced group that represented an intermediate outcome (Fig. 3a), worse than LumA but better than LumB, Basal and HER2E subtypes.

**Table 1:** Comparison of subtype classification between iRDM and PAM50 in four breast cancer datasets

	Method	Subtypes	Basal	HER2E	Immuno	Lum A	Lum B	Normal	Mixed*	Total
AFFY1951	iRDM	counts	310	211	342	441	351	209	87	1951
		percent	16%	11%	18%	23%	18%	11%	4%	100%
	PAM50	counts	361	244	NA	490	368	232	256	1951
		percent	19%	13%	NA	25%	19%	12%	13%	100%
METABRIC	iRDM	counts	298	208	383	425	387	199	97	1997
		percent	15%	10%	19%	21%	19%	10%	5%	100%
	PAM50	counts	339	255	NA	428	410	253	312	1997
		percent	17%	13%	NA	21%	21%	13%	16%	100%
TCGA	iRDM	counts	187	113	171	194	244	181	50	1140
		percent	16%	10%	15%	17%	21%	16%	4%	100%
	PAM50	counts	207	140	NA	262	256	171	104	1140
		percent	18%	12%	NA	23%	22%	15%	9%	100%
TARGETSEQ	iRDM	counts	112	49	80	120	55	41	26	483
		percent	23%	10%	16%	25%	11%	9%	5%	100%
	PAM50	counts	128	64	NA	134	67	33	57	483
		percent	26%	13%	NA	28%	14%	7%	12%	100%

The samples in the iRDM Immunity-enhanced group were further classified using the PAM50 algorithm. In the AFFY1951 training dataset there were 342 high-immunity tumors which were further separated into 14% Basal, 14% HER2E, 15% LumA, 27% LumB, 3% Normal, and 27% Mixed (Supplementary Table 4). Samples classified as Mixed displayed <95% confidence for assignment to a subtype. As shown in the heatmap (Supplementary Figure 5), expression of the 17 immunity genes was high in the Immunity-enhanced group compared to other PAM50 subtyping genes which were expressed at lower levels if expressed at all.

Similar to the calculated risk of recurrence (ROR) score using PAM50, the iRDM score was calculated using an immunity score in addition to subtype and proliferation scores to adjust the risk of distant metastasis. The iRDM score was calculated by two equations depending on ER and proliferation status, as described in the Materials and Methods section. As seen with the PAM50 assay (Fig. 3d), iRDM also divided patients into three risk groups with low, intermediate (med) and high risk of distant metastasis. The three risk groups demonstrated significant (*p* < 0.0001, *n* = 1951) outcome prediction in the training dataset AFFY1951 (Fig. 3c). Considering the impact of immunity genes on DMFS, iRDM significantly adjusted more patients into the lower risk category (Fig. 3c,d).

In both PAM50 and iRDM algorithms in this study, samples with low confidence (confidence < 0.95) were classified into a “Mixed” group (Table 1). A Mixed sample was not treated as a subtype as its gene expression pattern did not correlate well with any defined subtype. The percentage of Mixed samples was significantly reduced using the iRDM assay when compared with PAM50 (Table 1). The Immunity-enhanced group accounted for about 40% of the Mixed group defined by PAM50 (Data not shown).

**Fig. 4:**
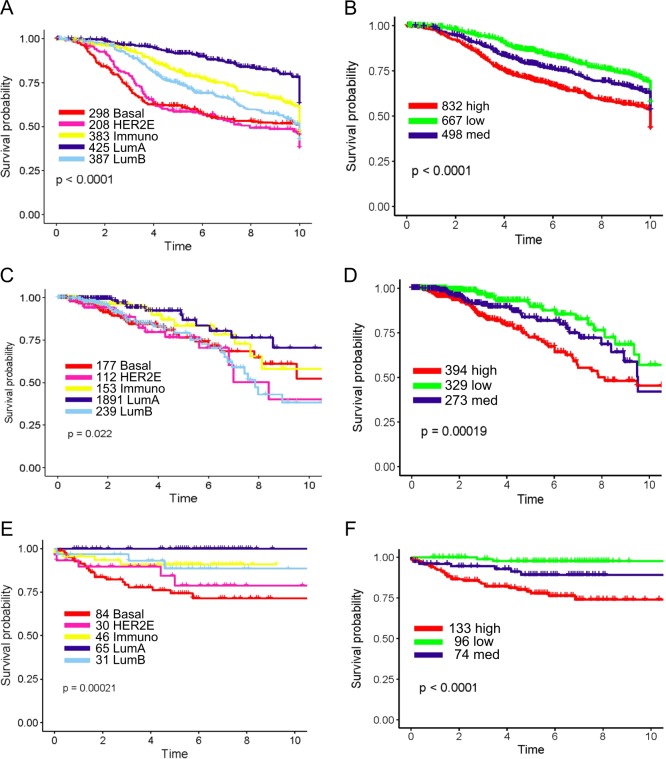
Validation of iRDM subtype and risk survival analysis in three independent test datasets. Survival plots of iRDM subtypes in METABRIC (A) (*p* < 0.0001, *n* = 1997), TCGA (C) (*p* = 0.022, *n* = 996), TARGETSEQ (E) (*p* = 0.00021, *n* = 303) and survival plots of risk groups (high, low, med) in METABRIC (B) (*p* < 0.0001, *n* = 1997), TCGA (D) (*p* = 0.00019, *n* = 996), TARGETSEQ (E) (*p* < 0.0001, *n* = 303) were shown. Subtypes and risk groups are color-coded: Basal-like (Red), HER2E (Hot Pink), Immuno (Yellow), Luminal A (Dark Blue), Luminal B (Sky Blue), Normal-like (Green); low (Green), med (Dark Blue), and high (Red) risks

The same results were observed in the three validation datasets, METABRIC, TCGA and TARGETSEQ (Fig. 4, Supplementary Figure 4) (Table 1). Overall survival (OS) was used as the outcome endpoint for the TCGA dataset and the results were slightly less significant in survival analysis compared to the other two datasets where DMFS was used.

One of the characteristics of the Claudin-low subtype (CLOW) of triple-negative breast cancers is high expression of immunity genes.^31^ To see if Immunity-enhanced and CLOW subtypes were the same, we monitored CLOW subtype tumors using expression of the top 80 CLOW signature genes.^31^ Less than 30% of the Immunity-enhanced tumors were classified as CLOW in the AFFY1951 dataset (Supplementary Figure 6A) although expression profiles of CLOW and Not-CLOW subgroups were very similar with a Pearson correlation of 0.97 (95% CI: 0.95–0.98, *p* < 0.00001). There was no prognostic difference between the CLOW and Not-CLOW subgroups (Supplementary Figure 6D) within the iRDM-defined Immunity-enhanced group. Similar results were observed in the METABRIC data with a Pearson correlation of 0.82 (95% CI: 0.74–0.88, *p* < 0.00001) and the TCGA data with a Pearson correlation of 0.92 (95% CI: 0.88–0.95, *p* < 0.00001) (Supplementary Figure 6) as two validation datasets. As the pattern of immunity gene expression in the CLOW tumors was the same as in the Not-CLOW tumors but of increased intensity, the CLOW subtype tumors appear to represent Immunity-enhanced tumors with the greatest level of immunity gene expression.

The immunity gene signature included B and T lymphocyte transcripts (Fig. 1) indicating a population of lymphocytes was present within breast cancers. To compare the gene expression-based immunity score with pathologist-assessed TIL, we retrieved 91H&E slides from triple-negative tumors with corresponding immunity scores (Supplementary Table 5). The average percentages of TIL in each tumor were determined by an experienced, licensed pathologist using an internationally recommended method^8^ (see also: www.tilsinbreastcancer.org). TIL percentages and immunity scores were significantly correlated (Pearson’s correlation = 0.75, 95%CI: 0.64 to 0.83, p value < 2.2E-16). As expected both TIL and immunity score predicted DMFS in the 91 patients (Supplementary Figure 7A & B). Patients with high TIL (>50% infiltrating lymphocytes) and high immunity scores had the best DMFS (Supplementary Figure 7C). Only two patients had high TIL and a low immunity score, and these patients are not shown. Among patients with low TIL ( < 50% infiltrating lymphocytes), the immunity score influenced outcome, with a high immunity score portending better DMFS than a low immunity score (Supplementary Figure 7C).

### Tumor heterogeneity within breast cancer subtype

**Fig. 5:**
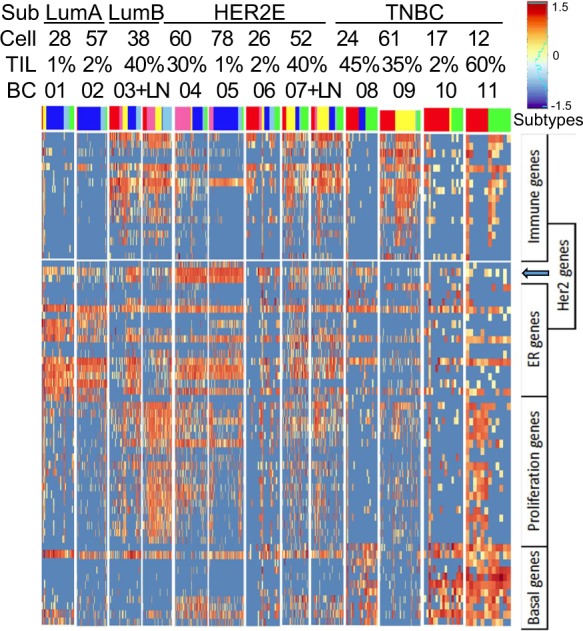
Heatmap of iRDM subtypes analyzed in 549 single cells from 11 primary breast tumors and two lymph node metastases. All tumors showed two or more iRDM subtypes. Sub, subtype; Cell, single cell number; TIL, percentage of tumor-infiltrating lymphocytes. Color-coded individual cell subtypes: Basal-like (Red), HER2E (Hot Pink), Immuno (Yellow), Luminal A (Dark Blue), Luminal B (Sky Blue), Normal-like (Green), Mixed (Black)

Single-cell expression of the 72 genes was also analyzed in 11 tumor samples containing 12 to 78 single cells.^27^ The subtype of each bulk tumor was defined by immunohistochemistry (IHC) in the original paper, identifying 2 LumA, 1 LumB, 4 HER2E and 4 triple-negative breast cancers (TNBC). We used iRDM algorithm to identify each cell’s subtype (Supplementary Figure 8). Single cell analysis indicated that tumor cells in each tumor displayed expression of at least two subtypes detected by iRDM (Fig. 5), showing heterogeneity of gene expression within a tumor. The correlation between the percentage of TIL estimated in the tumor and immunity gene expression was of intermediate strength. In most cases, tumors with a high percentage of TIL had higher expression of immunity genes and tumors with a low percentage of TIL had lower or no expression of immunity genes. However, there was a tumor with high TIL and low immunity gene expression (BC08) and a tumor with low TIL and high immunity gene expression (BC06). Immunity gene expression did not appear to coincide with expression of basal cytokeratins or signals from HER2(ERBB2) and steroid receptors, implying that the expression of immunity genes was not correlated with breast cancer subtypes (Fig. 5).

## Discussion

Recent studies have identified genes that influence anti-tumor immunity.^3–6,10,32,33^ In this paper, we identified 17 co-expressed immunity genes that, as a group in gene ontology analysis, play an important role in immune response and its regulation. The Immunity-enhanced group identified in this analysis consisted in part of previously unclassified tumors with relatively low expression of the PAM50 intrinsic subtype marker genes. It also included the Claudin-low subtype of triple-negative breast cancers. We defined Immunity-enhanced patients as a new prognostic group instead of a subtype due to subsequent data analysis in single cells showing that the majority of immunity gene expression was contributed by infiltrating immune cells in tumors. The iRDM algorithm improved the accuracy for breast cancer classification by significantly reducing the frequency of unclassified Mixed tumors in four independent datasets. Overexpression of the 17 immunity genes was found to be predictive of a good or better prognosis, meaning a lower risk of cancer recurrence, metastasis or death. This observation supports others’ prognostic analyses of breast cancer and ovarian cancer.^10,34^ In the current study, one important finding was immunity genes play a significant role in breast cancer prognosis only in patients whose tumors are estrogen-negative and highly proliferative, accounting for about 24% of all patients in our training dataset. This was corroborated by gene expression analysis of tumor single cells showing that expression of proliferation genes can sometimes be contributed by active TIL instead of carcinoma cells. To estimate more precisely the risk of distant metastasis for these patients, it is necessary to incorporate an immunity score, besides the intrinsic subtype and proliferation scores as used in the PAM50 assay, into the equation for calculation of risk.

The Immunity-enhanced group in breast cancer was identified by adding a set of 17 co-expressed immunity genes into the PAM50 marker genes. Interestingly, Immunity-enhanced group tumors had high expression of genes that were up-regulated in Claudin-low subtype tumors. We considered whether the Claudin-low subtype was similar to the Immunity-enhanced group as both have high immunity gene expression and low or no intrinsic subtyping gene expression. Only one third of tumors in the Immunity-enhanced group expressed the Claudin-low signature genes above the cutoff threshold for the Claudin-low subtyping algorithm.^31^ However, the Claudin-low and not-Claudin-low subtypes within the Immunity-enhanced group were highly correlated and had the same prognosis. Thus, we conclude that Claudin-low subtype tumors represent Immunity-enhanced group tumors with the greatest levels of immunity gene expression. It is notable that anti-tumor immunity within CLOW tumors appears to include immune-suppressive T lymphocytes.^35^

For tumors with high TIL and low expression of HER2 (ERBB2), ER, PR, basal keratins and proliferation genes, the PAM50 classification spreads them across five subtypes but predominantly in the uncertain Mixed category. The presence of the immune cells does not appear to affect expression of the other PAM50 markers as, for the most part, there was no correlation between high immunity gene expression and any of the other marker genes (Fig. 1B and Supplementary Figure 4). When the PAM50 algorithm was used to cluster genes including the 17 immunity genes, there was a clear gradation of immunity in all subtypes and generally without gradation of the other markers within each subtype. This suggests that Immunity-enhanced tumors are not a subtype within a PAM50-defined subtype, but a distinct group of breast cancers, similar to the Immunity-enhanced groups in melanoma and ovarian cancer.^36^

The lack of a strong correlation between immunity gene expression and the other molecular markers indicates that the low expression of PAM50 markers in the Immunity-enhanced group was not due to dilution of tumor mRNA with infiltrating lymphocytes. The major reason why immunity genes were not observed in the intrinsic subtype analysis is the algorithm filters out variable genes, such as the highly variable immunity genes, within a subtype. There appears to be a distinct subset of breast cancers with low expression of the PAM50 markers and high expression of immunity genes.

The Immunity-enhanced group of breast cancer with relative low expression of ER, PR, HER2, basal cytokeratins and proliferation drivers might be less responsive to treatments with anti-estrogen, Herceptin and general chemotherapies, but may benefit more from the immunotherapies. The immunity score could be a companion diagnostic maker in addition to PDL-1 expression for immune-checkpoint inhibitors. A clinical trial that stratifies patients based on subtypes including the Immunity-enhanced group may distinguish patients with high anti-tumor immunity from those with low anti-tumor immunity and provide more precise design of trials testing the efficacy of immune-checkpoint inhibitors.

Several clinical trials have demonstrated that number of TIL are prognostic in breast cancers.^8,9,37,38^ Pathologic TIL on H&E slides were significantly correlated with immunity scores based on our analysis of 91 triple-negative breast cancers. Our analysis of single-cell RNA-seq data indicated that expression of the immunity genes was contributed mainly by immune cells, not carcinoma cells, suggesting that expression levels of immunity genes may reflect the number of TIL. Single-cell analysis also indicated that only a portion of immune cells, not all TIL, were proliferative in some solid tumors. We speculate that only the proliferative TIL are active in anti-tumor immunity and this may explain why some patients having high TIL had poor prognosis in clinical studies.^33,34^

Tumor heterogeneity was also studied by analyzing expression of the 72 genes in single tumor cells. Of the 11 tumors analyzed in the current study, each tumor had at least two intrinsic subtypes. This observation needs further validation by conducting large-scale single-cell RNA-seq of solid tumors using advanced technologies such as 10x Genomics to evaluate its value in clinical design of adjuvant therapies for breast cancer patients.

In summary, a set of immunity genes was extracted through analysis of a large dataset of breast cancers. High expression of the immunity genes identified an Immunity-enhanced group and indicated a better prognosis in ER-negative and high-proliferation breast cancers. Single-cell sequencing provides a useful tool for mechanistic studies of tumor immunity and heterogeneity with more studies needed to evaluate its clinical value.

## Methods

### Patients

This study included 225 anonymous patients from a multi-center study in Shanghai and 250 patients from the University of North Carolina at Chapel Hill (UNC-CH). 225 breast tumor FFPE blocks were obtained from patients hospitalized and receiving modified radical mastectomy or lumpectomy in Shanghai Cancer Hospital. The patient-anonymous 250 FFPE tissues were obtained from UNC Hospital and were part of a molecular epidemiology study, LCCC-9830. The study was approved by the two independent institutional review boards (IRB) at the Shanghai Tumor Institute at Fudan University and UNC at Chapel Hill.

### Microarray data mining and analysis

Affymetrix microarray data sets for 2034 patients from fourteen breast cancer cohort studies were retrieved from GEO: GSE1112, GSE12093, GSE1456, GSE2034, GSE2603, GSE3494, GSE4922, GSE5327, GSE6532, GSE7378, GSE7390, GSE8193, GSE9195, and ArrayExpress|E-TABM-158.^39,40^ The downloaded individual CEL files were first processed by Robust Multi-chip Average^41^ and then merged into one dataset of 2034 expression profiles which were further batch-corrected using Combat^42^ with subtype as covariate. An unsupervised analysis of the 2034 expression profiles using the pattern-recognition algorithm EPIG^43,44^ was performed to identify sets of co-expressed genes. Two co-expressed gene clusters with significant enrichment of gene ontology categories “Immune Response” and “Cell Cycle” were identified (Supplementary Table 2). 119 immunity and 71 cell cycle genes were consistently selected in 1000 iterations by EPIG, in which 80% of the 2034 expression profiles were randomly selected in each iteration. Gene numbers were subsequently reduced by selecting the top-weighted EPIG values, by correlation with the 119 immunity genes (R > 0.87, Figure 8), and by using gene ontology to eliminate duplicate functional genes. A compact 17 immunity gene signature was generated containing *APOBEC3G, CCL5, CCR2, CD2, CD27, CD3D, CD52, CORO1A, CXCL9, GZMA, GZMK, HLA-DMA, IL2RG, LCK, PRKCB, PTPRC*, and *SH2D1A*. We used the same methods to identify 19 proliferation genes including *AURKA, BIRC5, CCNB1, CCNE1, CDC20, CDC6, CENPF, CEP55, EXO1, MKI67, KIF2C, MELK, MYBL2, NDC80, ORC6, PTTG1, RRM2, TYMS, and UBE2C*. 1951 patients of the 2034 patients had follow-up and clinical data which were combined using the same established method as described^45^ and this group was named “AFFY1951”.

In addition to the AFFY1951 training dataset, we also assembled three test datasets on different platforms including Illumina beads arrays of 1997 fresh-frozen breast tumors (METABRIC),^28^ RNA-seq of 1140 fresh-frozen breast tumors (TCGA)^46^ and targeted RNA-seq of breast cancer FFPE tissues from 225 samples from Shanghai Cancer Hospital and 258 samples (250 patients with 8 duplicate samples) from UNC-CH (TARGETSEQ, GSE113863).

### Calculation of Proliferation and Immunity Scores

A proliferation score was calculated by averaging expression levels of the afore-mentioned 19 proliferation genes as “unscaled proliferation score” in a sample and then scaled between 0 and 100 using the formula: 38 × (unscaled proliferation score + 1.35). Proliferation-high were those samples with proliferation score larger or equal to 50 while proliferation-low were the others with score less than 50.

An Immunity score was calculated by averaging gene expression values of the above 17 immunity genes as “unscaled immunity score” and then scaled between 0 and 100 for each sample using the formula: 30 × (unscaled immunity score + 1.4). For Immunity score group classification, the patients were divided into two groups, “iweak” and “istrong”, based on their Immunity score values using the cut-off value of 42 that was derived from the combined data using X-tile.^47^

### Breast Cancer Molecular Subtyping

A panel of 72 genes, consisting of the 17 immunity genes, 19 proliferation genes, 11 Basal genes, 14 ER genes, 3 HER2E genes, 2 invasion genes, and 6 housekeeper genes was formed for analysis of breast cancer subtypes (Supplementary Table 3). Ten-fold CV included different statistical predictors including PAM,^48^ a k-Nearest Neighbor Classifier (KNN) with either Euclidean distance or one-minus-Spearman-correlation as the distance function and a Class Nearest Centroid (CNC) metric with either Euclidean distance or one-minus Spearman-correlation as the distance function. A sample was assigned the subtype corresponding to the highest one-minus Spearman-correlation value among the six values versus centroids for iRDM subtypes: Basal, HER2E, Immuno, LumA, LumB, and Normal based on Single Sample Predictor algorithms for subtyping breast cancer.^23,29,49^

Confidence intervals for each subtype identification were calculated^50^ and a subtype with confidence lower than 95% was called “Mixed”. The formula for confidence calculation is: Subtyping “confidence” = 1 - Spearman’s test p value.

Survival plots were done using R package Survminer (downloaded from Bioconductor “RTCGA”) which provided censored survival curves. In addition, Univariate Kaplan-Meier survival analysis was performed for validation using WINSTAT for EXCEL^®^ (R. Fitch Software, Lehigh Valley, Pa.).

### The iRDM prediction model

Factors included in the model to optimize an outcome predictor were molecular subtype, proliferation score and immunity score that were calculated based on expression profiles of the 72-gene panel. We slightly modified established algorithm^23^ for iRDM and used Cox models for iRDM score calculation in which Distant Metastasis-Free Survival Time (DMFS) was used with patient follow-up for up to 10 years. A subset of 404 patients was selected as a training dataset through ranking of correlation to the centroids with a cutoff at 0.7. Coefficients for each subtype were calculated using this Cox model and used as constant factors for subtype Spearman correlations, proliferation score, and immunity score. The immunity-stratified Risk of Distance Metastasis (iRDM) was calculated using two formulas:

A. For the proliferation-high and ER-negative group only:
  $$\begin{array}{*{20}{l}} {{\mathrm{Unscaled}}\,{\mathrm{iRDM}}\,{\mathrm{score}}} \hfill & = \hfill & { - (0.02 \times {\mathrm{Basal}}) + 0.16 \times {\mathrm{HER2E}} + ( - 0.34 \times {\mathrm{Immuno}})} \hfill \\ {} \hfill & {} \hfill & { + 0.07 \times {\mathrm{LumA}} + 0.08 \times {\mathrm{LumB}} + 0.09 \times {\mathrm{Proliferation}}\,{\mathrm{Score}}} \hfill \\ {} \hfill & {} \hfill & { + ( - 0.40 \times {\mathrm{Immunity}}\,{\mathrm{Score}})} \hfill \end{array}$$
B. For all other groups except A:

$$\begin{array}{*{20}{l}} {{\mathrm{Unscaled}}\,{\mathrm{iRDM}}\,{\mathrm{score}}} \hfill & = \hfill & {0.40 \times {\mathrm{Basal}} + 0.48 \times {\mathrm{HER2E}} + \left( { - 0.06 \times {\mathrm{Immuno}}} \right)} \hfill \\ {} \hfill & {} \hfill & { + \left( { - 0.46 \times {\mathrm{LumA}}} \right) + 0.19 \times {\mathrm{LumB}} + 0.24} \hfill \\ {} \hfill & {} \hfill & { \times {\mathrm{Proliferation}}\,{\mathrm{Score}} + \left( { - 0.08 \times {\mathrm{Immunity}}\,{\mathrm{Score}}} \right)} \hfill \end{array}$$

The unscaled iRDM score was further scaled to values spanning 0 to 100 by the formula:

$${\mathrm{iRDM}}\,{\mathrm{score}} = 90 \times {\mathrm{Unscaled}}\,{\mathrm{iRDM}}\,{\mathrm{score}} + 50$$

Patients were categorized into three groups, low, intermediate (med) and high risk, according to iRDM scores (range 0–100) with cutoffs at 33 and 50 optimized by X tile.^47^

### Targeted RNA Expression by RNA-seq

For breast tumor FFPE tissues RNA extraction was routinely performed using Roche FFPE RNA extraction kit according to the manufacturer’s protocol. The Illumina TruSeq Targeted RNA expression kit was used to build libraries of the targeted 72-genes. To synthesize cDNA, 200 to 800 nanograms of purified FFPE RNA in a total volume of 3 µl was mixed with 4.0 µl RCS1, 2.0 µl ProtoScrip II Reverse Transcriptase, 1.0 µl 10 mM DTT at 42 °C for 30 min and 94 °C for 10 min. The cDNA was hybridized with custom oligo pools in a thermal cycler programed to gradually decrease temperature from 70 °C to 30 °C in 30 min. The RNA/Oligo hybrid products were washed, extended and ligated. The ligated DNA was amplified by DNA polymerase on the thermal cycler with 35 PCR cycles of 98 °C for 30 s, 62 °C for 30 s and 72 °C for 60 s. The PCR products were purified with AMPure XP beads and eluted in 15 µl of buffer, measured using Agilent Bioanalyzer2100 and DNA1000 chips, pooled with equal amounts of DNA from each sample’s library, and finally diluted to 4 nM, denatured, and loaded to NextSeq 500 according to the manufacturer’s protocol. Illumina Casava1.7 software was used for basecalling and sequencing data were demultiplexed with Illumina bcl2fastq2 software to generate one fastq file per sample. To ensure sequencing data integrity of libraries derived from FFPE RNA tissues, only samples with total reads larger than 10000 and missing genes less than 30% of all 72 genes were further processed in the validation study. Single read 1 sequence in each fastq file were mapped to known targeted regions of human genomes to generate raw counts using R package ShortRead. Raw counts of all samples were normalized by the size of the transcripts and by the size of the library and then calculated for CPM per sample as a gene expression matrix using R package edgeR from Bioconductor and finally log based 2 transformed and imputed by KNN method. Gene expression data were further median-centered and column-standardized. We also used Distance Weighted Discrimination DWD^51^ to combine 225 Targeted RNA samples from Shanghai Cancer Hospital and 258 Targeted RNA samples including 8 technical repeats from UNC-CH to create a test dataset denoted “TARGETSEQ” (GSE113863) in which 303 patients had DMFS time and event (Supplementary Table 4).

### Histopathological TIL-assessment

The TILs were evaluated by a trained pathologist using an internally recommended method.^8^ See also www.tilsinbreastcancer.org for a freely available training tool for the assessment of TILs by pathologists on HE-slides.

### Study approval and consent to participate

All studies were carried out according to institutional guidelines, and with appropriate informed consent from participants. Institutional ethics committees of the clinical centers where samples were collected reviewed and approved all protocols. The Institutional Review Board of the Shanghai Cancer Center at Fudan University and University of North Carolina separately approved procurement and handling of the human materials. All data were analyzed anonymously.

## Electronic supplementary material

Supplemental Figures and Tables

## Data Availability

Original primary sequencing data and processed data for Targeted RNA-seq data TARGETSEQ created in this study were submitted to GEO sequencing data (GSE113863) at https://www.ncbi.nlm.nih.gov/geo/query/acc.cgi?acc=GSE113863. Published data for generating training data AFFFY1951 were retrieved from GEO: GSE1112, GSE12093, GSE1456, GSE2034, GSE2603, GSE3494, GSE4922, GSE5327, GSE6532, GSE7378, GSE7390, GSE8193, GSE9195, and ArrayExpress|E-TABM-15839,40. METABRIC gene expression data are available at the European Genome-Phenome Archive http://www.ebi.ac.uk/ega/ which is hosted by the European Bioinformatics Institute, under accession number EGAS00000000083. TCGA breast cancer sequencing data are available in CGHub (https://cghub.ucsc.edu/) and sample lists, data matrices and supporting data can be found at (http://tcga-data.nci.nih.gov/docs/publications/brca_2012/). A RNA-seq dataset of 2,700 Peripheral Blood Mononuclear Cells (PBMC) single cells is freely available from 10X Genomics that were sequenced on the Illumina NextSeq 500 and the raw data can be found on http://satijalab.org/seurat/pbmc3k_tutorial.html. TPM counts of eleven solid breast tumors’ single cell RNA-seq can be downloaded from GEO (GSE75688) https://www.ncbi.nlm.nih.gov/geo/query/acc.cgi?acc=GSE75688.

## Electronic supplementary material

**Supplementary Information** accompanies the paper on the *npj Breast Cancer* website (10.1038/s41523-018-0088-0).
