# Supplementary material for: An assessment of prognostic immunity markers in breast cancer
Source: NPJ Breast Cancer. 2018 Oct 29;4:35. doi: 10.1038/s41523-018-0088-0 (PMC6206135; doi:10.1038/s41523-018-0088-0)
Supplement: Supplementary file 1 — Supplemental Figures and Tables [file 41523_2018_88_MOESM1_ESM.pdf]

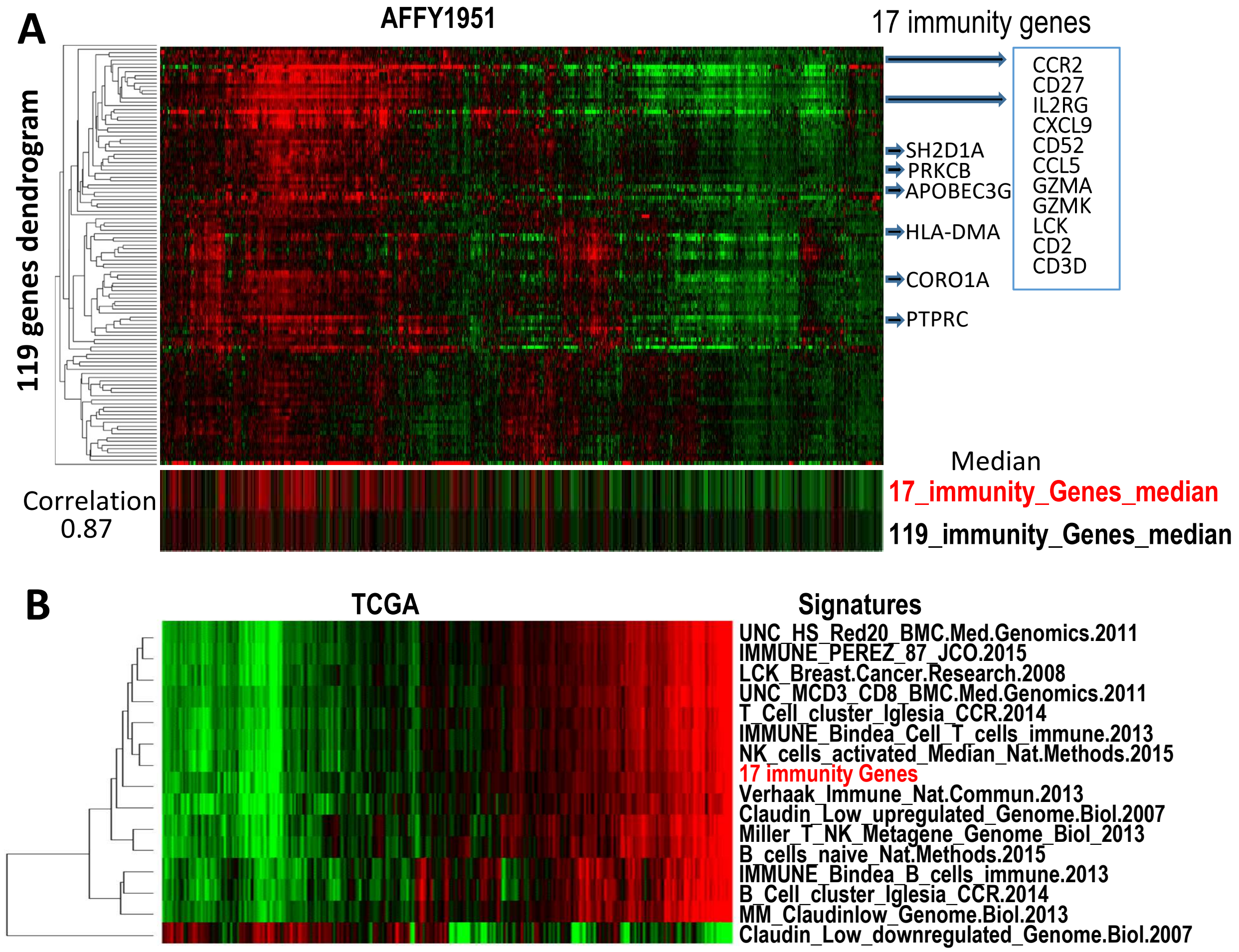

Supplementary Figure 1. Selection of 17 immunity genes from 119 significant immunity genes by EPIG and the correlation among 17 immunity gene signature and other published immunity signatures . (A) Distribution of the 17 immunity genes among the significant immunity genes. The correlation between the 17 immunity genes and 119 immunity genes was 0.87 (95%CI 0.86-0.88). (B) The 17 immunity genes in this study were positively correlated with 13 published immunity signatures, including UNC Claudin-low subtype up-regulated, with Person correlations between 0.88 and 0.94, but negatively correlated with UNC Claudin-low subtype down-regulated gene signature (-0.51, 95%CI -0.55 to -0.46).

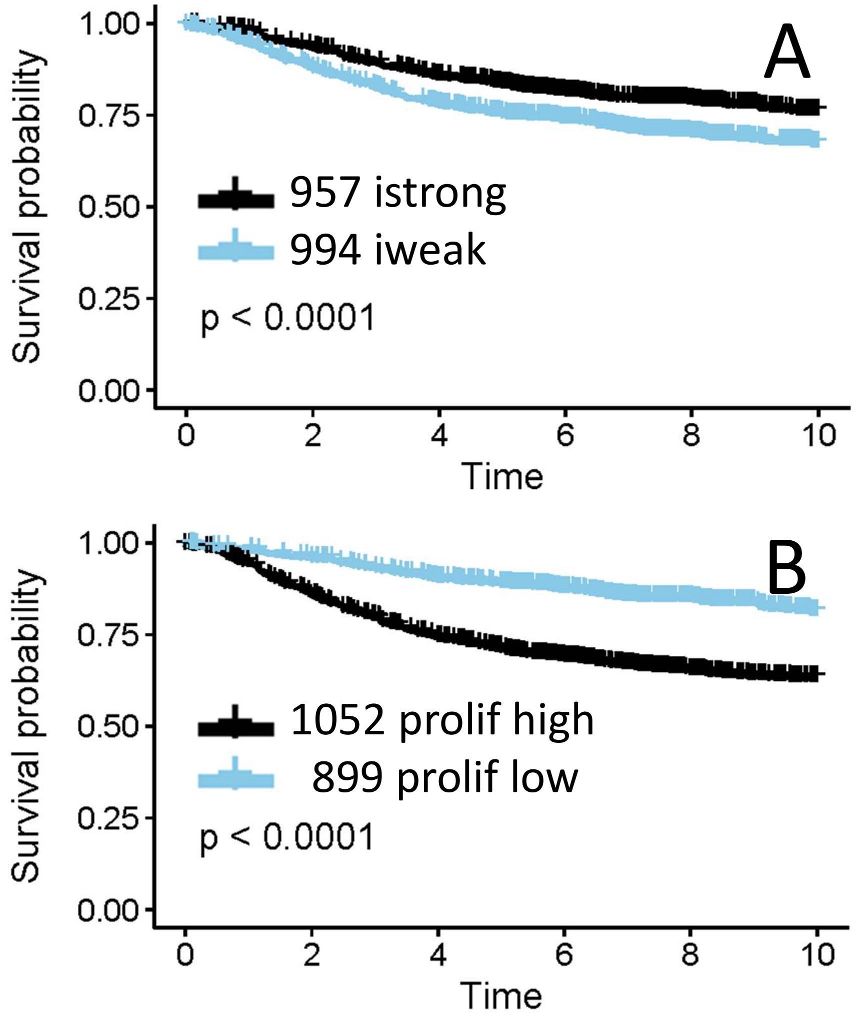

Supplementary Figure 2. Survival analysis of the immunity-strong group (istrong) and the immunity-weak group (iweak) (A), and proliferation high group and proliferation low group (B) in AFFY1951.

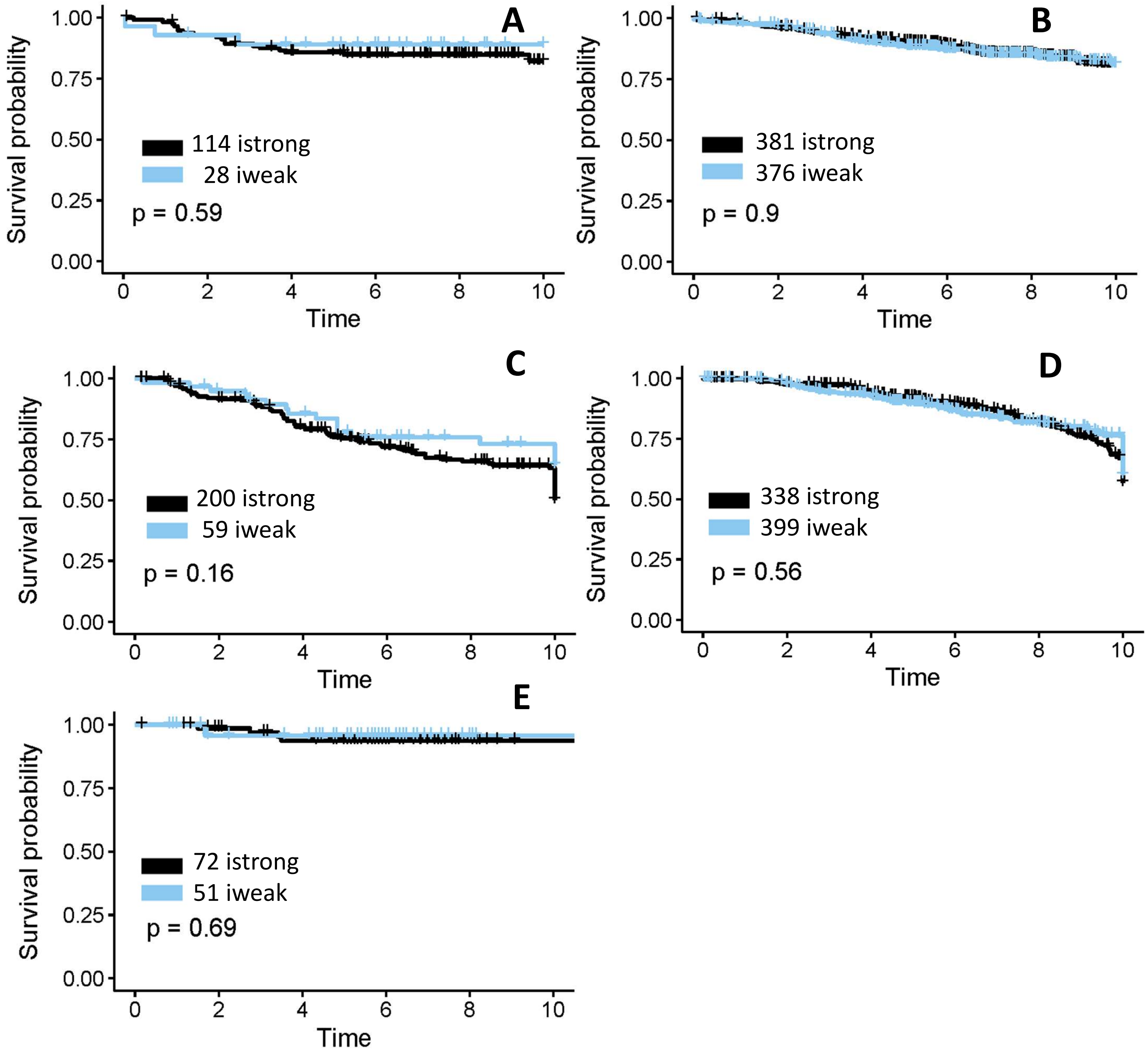

Supplementary Figure 3. Survival plots of Immunity Score in different patient groups identified by low proliferation only and ER status in three datasets. Immune Score demonstrated insignificant prognosis in low proliferation group in AFFY1951 ER-negative (A) (p=0.59, n=142) or ER-positive (B) (p=0.9, n=757) , METABRIC ER-negative (C) (p=0.16, n=259) or ER-positive (D) (p=0.56, n=737), and TARGETSEQ ER-positive E) (p=0.69, n=123). Data was not shown for ER-negative and proliferation low group in TARGETSEQ dataset due to the low number of patients (n=18 with only 1 istrong) in this group.

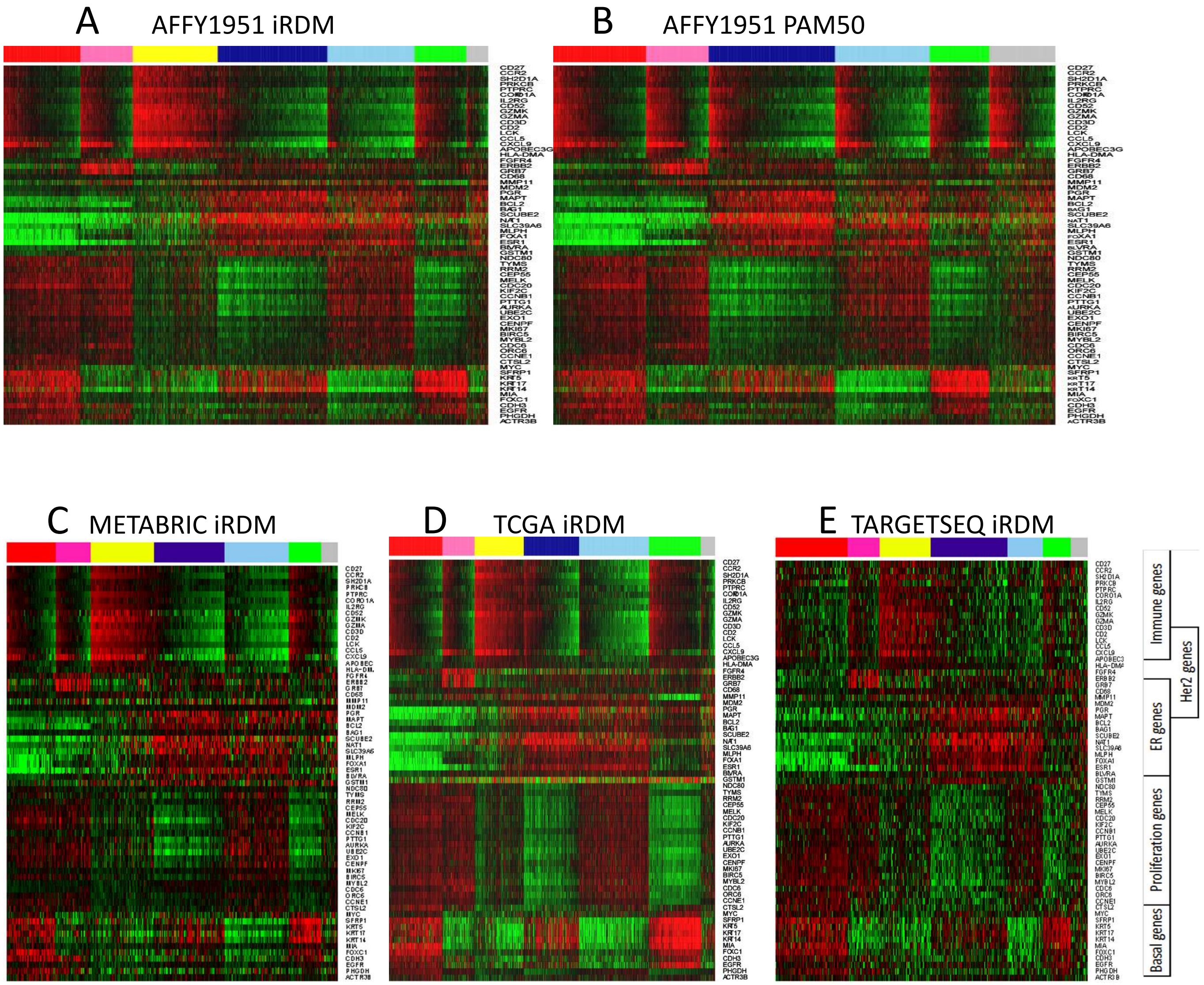

Supplementary Figure 4. Comparison of heatmaps of iRDM (A) and PAM50 (B) in AFFY1951 breast cancer training dataset and validation of iRDM subtypes in three independent test datasets METABRIC (C), TCGA (D), and TARGETSEQ (E). Subtypes are shown by color-coded bars: Basal-like (Red), HER2E (Hot Pink), Immuno (Yellow), Luminal A (Dark Blue), Luminal B (Sky Blue), Normal-like (Green), Mixed (Grey, not a subtype but a collection of samples with subtype confidence less than 0.95). Immuno group were observed across all four independent datasets using different platforms.

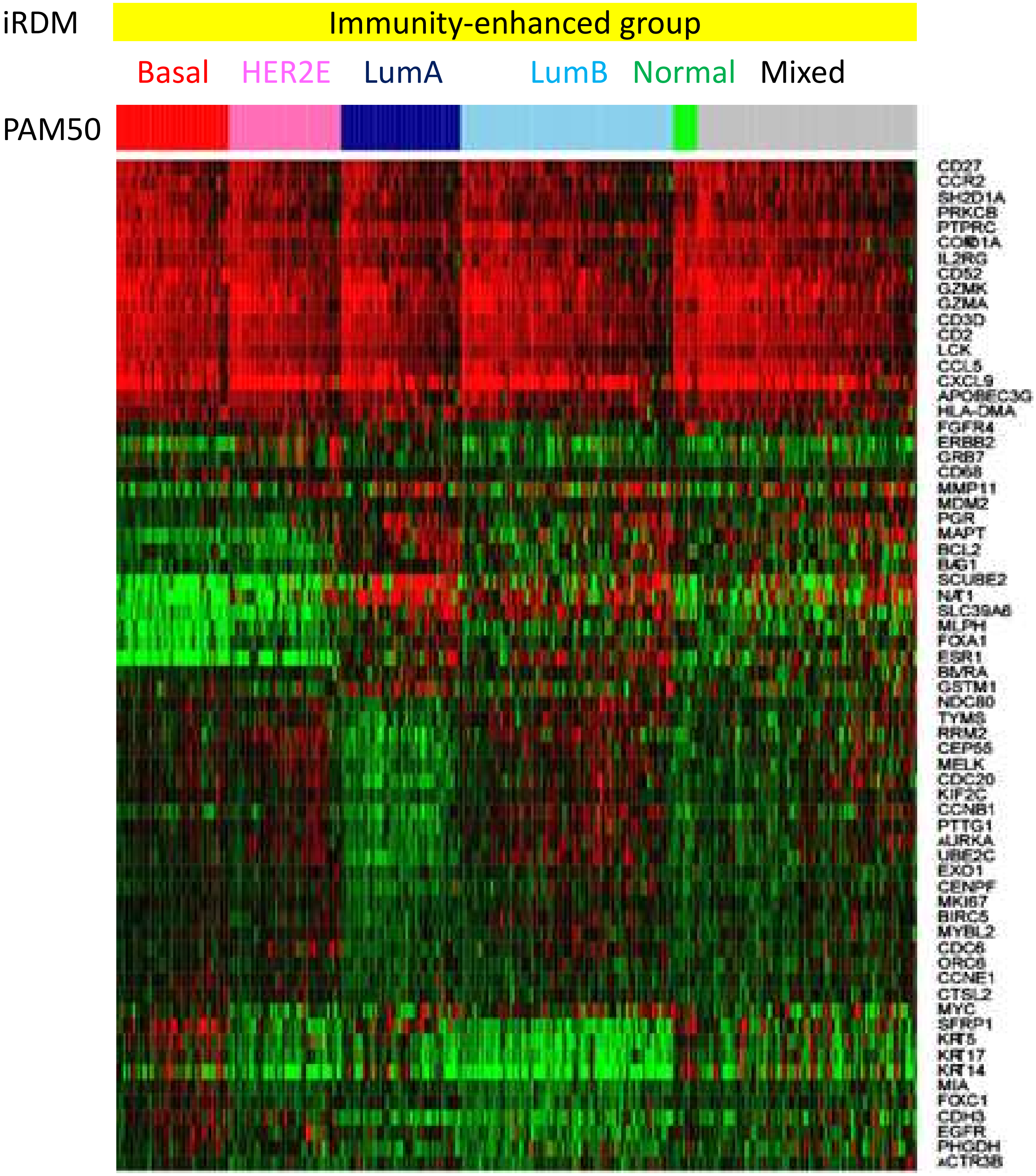

Supplementary Figure 5. Heatmap of the Affy1951 iRDM Immunity-enhanced group samples only (N=342) with samples ordered by PAM50 subtypes. PAM50 subtypes were color-coded. Gene clusters in the heatmap demonstrated that the 17 immunity genes were highly expressed in all PAM50 subtypes.

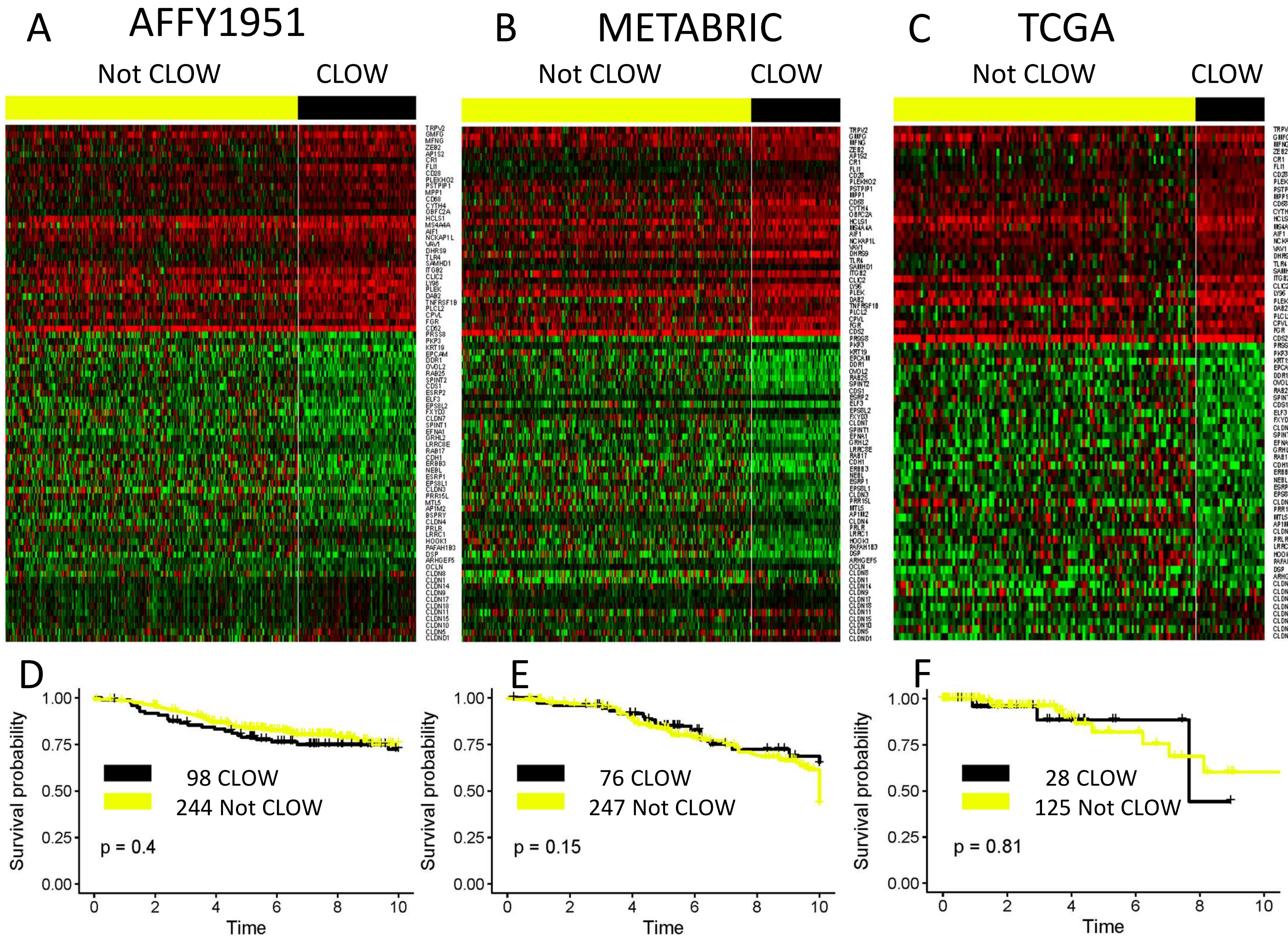

Supplementary Figure 6. Heatmap of the Claudin-low (CLOW) signature genes in the Immunity-enhanced subtype. Expression of the top 80 Claudin-low signature genes (Prat et al, BCR 2010)(fix this reference) identified less than 1/3 of Immunity-enhanced subtype samples as CLOW subtype (black color bar) in each of the three datasets (A-C). Not-CLOW (yellow color bar) and CLOW groups within the Immunity-enhanced subtype were highly correlated and had no difference in survival prognosis in AFFY1951 (D) (p=0.4, n=342), METABRIC (E) (p=0.15, n=323), TCGA (F) (p=0.81, n=151).

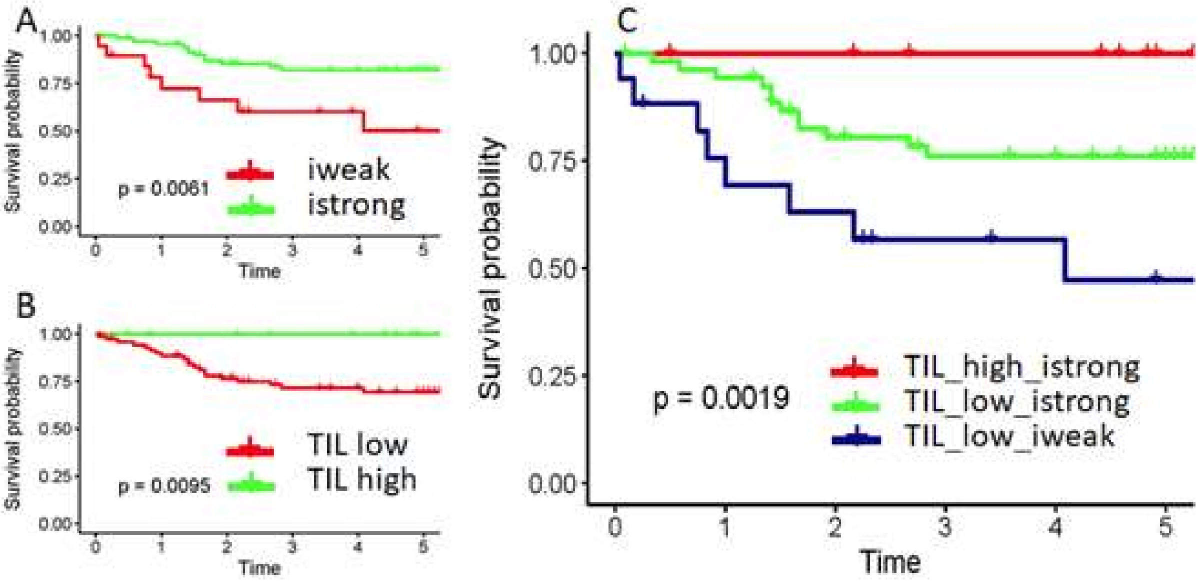

Supplementary Figure 7. Comparison of survival analysis with TILs percentage and immunity score. Kaplan-Meier plots were used to show Distant Metastasis-Free Survival (DMFS) based on immunity score only (A) (p=0.0061, n=91), TILs percentage only (B) (p=0.0095, n=91) and 3 groups of TILs and immunity scores (C) (p=0.0019, n=89, Note: the 2 patients with "TIL_high_iweak" were excluded in this plot.

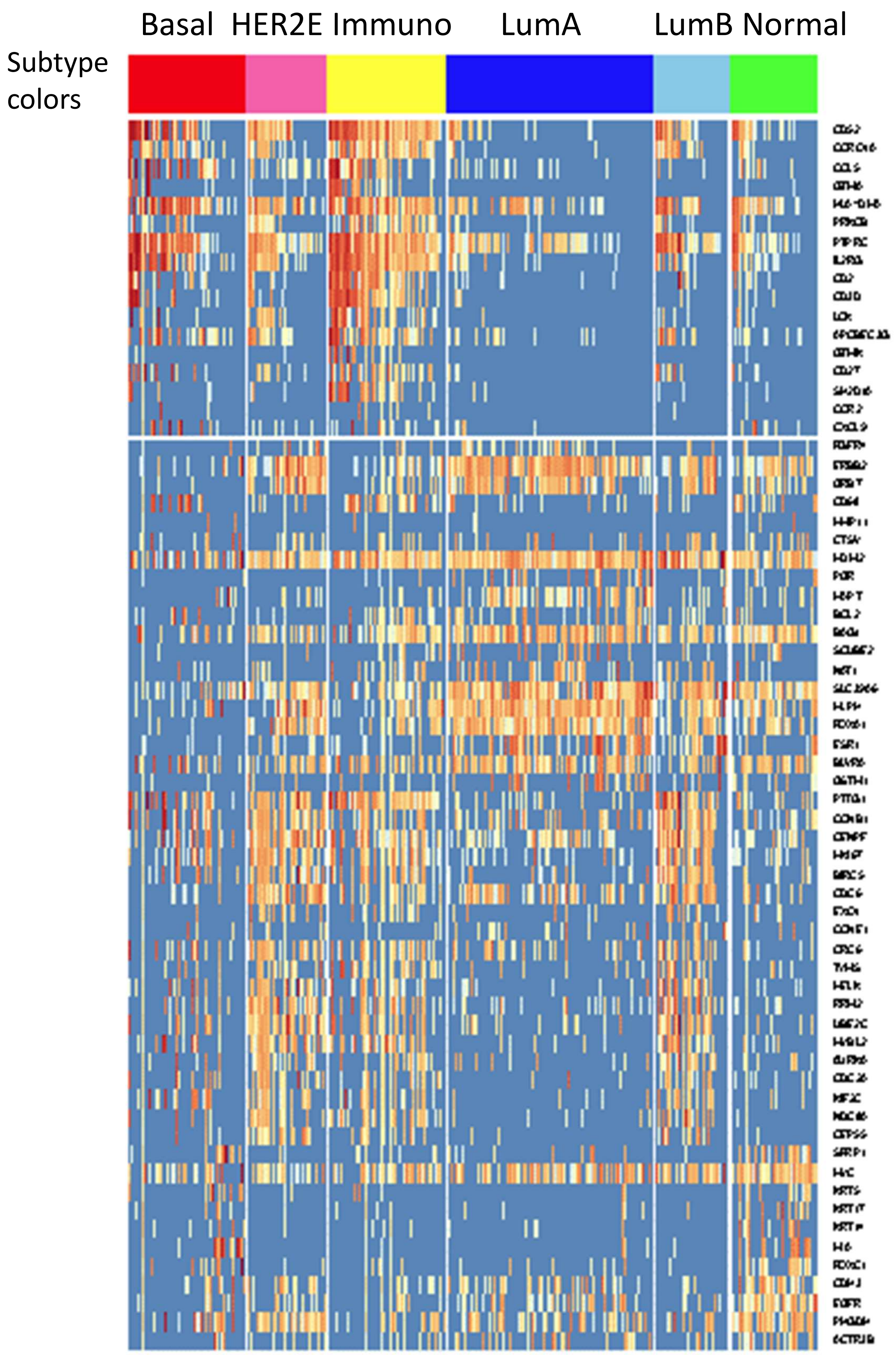

Supplementary Figure 8. Heatmap of individual cell's subtype using iRDM classification algorithm in which Mixed subtype was omitted. Color-coded individual cell subtypes: Basal-like (Red), HER2E (Hot Pink), Immuno (Yellow), Luminal A (Dark Blue), Luminal B (Sky Blue), Normal-like (Green).

Supplemental Table 1. Clinical characteristics of training dataset AFFY1951 breast cancer patients.

		AFFY1951	
		**No.**	**%**
**Age years**		56.8±13.2	
	<50	527	27%
	⩾50	1029	53%
	NA	395	20%
**Lymph node**	Negative	1495	77%
	Positive	437	22%
	NA	19	1%
**Grade**	I	235	12%
	II	652	33%
	III	576	30%
	NA	488	25%
**Size**	⩽2cm	986	51%
	>2cm	766	39%
	NA	199	10%
**ER**	Negative	399	20%
	Positive	1342	69%
	NA	210	11%
**HER2**	Negative	NA	NA
	Positive	NA	NA
**Chemotherapy**	Yes	281	14%
	No	1655	85.00%
	NA	15	1%

Suplemental Table 2.
EPIG significant 119 immunity genes

119 genes	average	cellHigh_immuLow_a	cellHigh_immuhigh_a	diff	score
CXCL9	8.881296067	7.705733512	9.883953748	2.17822	42.13858
CCL5	7.527790167	6.702478966	8.239709729	1.537231	17.78876
CD52	6.860428024	6.048098039	7.536509091	1.488411	15.19837
PTPRC	7.104135005	6.323024777	7.758551834	1.435527	14.63976
CD2	7.632617896	6.902300357	8.234991547	1.332691	13.55603
GZMK	7.328120452	6.594784135	7.914896651	1.320113	12.77069
CD3D	7.579391052	6.896737433	8.146519458	1.249782	11.83867
LCK	6.771085644	6.082288235	7.316821691	1.234533	10.31963
GZMA	6.117603245	5.419224777	6.671079107	1.251854	9.587136
IL2RG	7.664152507	7.07873672	8.143615311	1.064879	8.690892
CORO1A	7.400114848	6.805701961	7.886459171	1.080757	8.643602
APOBEC3G	6.854390069	6.297092513	7.299986922	1.002894	6.894126
CCR2	5.820885349	5.273706952	6.259633174	0.985926	5.658195
CD27	6.532586627	6.030417112	6.919514673	0.889098	5.163974
PRKCB	6.155890954	5.660757754	6.54393445	0.883177	4.801602
SH2D1A	5.573901672	5.407783244	5.694282137	0.286499	0.457515
**HLA-DMA**	8.704678073	8.675562032	8.741313078	0.065751	0.037632
TRBC1	7.795880629	6.821183244	8.603487879	1.782305	24.76447
LYZ	9.321175221	8.572738324	9.993427911	1.42069	18.81348
CCL19	7.577187119	6.754179679	8.25994992	1.50577	17.18009
XCL1	5.56903235	4.644604456	6.323886443	1.679282	15.7046
LAMP3	6.519647099	5.810331194	7.110577352	1.300246	11.02238
SLAMF8	6.586710619	5.932955258	7.138169537	1.205214	9.56747
CD48	7.988642085	7.405241711	8.47075949	1.065518	9.06973
C1QA	8.56562527	8.023713904	9.036531738	1.012818	8.786618
IL7R	7.25845703	6.669098752	7.752551356	1.083453	8.520482
LTB	7.202739626	6.627903922	7.685364753	1.057461	8.054272
SELL	6.452876106	5.824157932	6.940019617	1.115862	8.034781
PLAC8	6.121699017	5.513721034	6.598760128	1.085039	7.207136
RAC2	8.840514258	8.342972014	9.237388357	0.894416	7.07224
CD8A	7.492474926	6.949113012	7.916800638	0.967688	7.016098
EVI2B	6.504481023	5.931199465	6.966726635	1.035527	6.974862
CYTIP	5.819824287	5.208809091	6.303160128	1.094351	6.969846
CSF2RB	6.682442675	6.126813547	7.147177671	1.020364	6.957378
HCLS1	7.587598623	7.065409804	8.018990431	0.953581	6.899525
IRF8	7.575857424	7.059678966	7.996013238	0.936334	6.64192
GZMB	5.337810324	4.734463815	5.79995327	1.065489	6.059844
CCR7	5.799630777	5.217302317	6.239142424	1.02184	6.055726
IL10RA	7.213887709	6.71096328	7.62658756	0.915624	6.047891
PLEK	7.271571288	6.78511533	7.66816571	0.88305	5.670211
TRAC	7.705717502	7.231694652	8.085469856	0.853775	5.616945
RUNX3	6.42473176	5.934492513	6.847080223	0.912588	5.350622
TRAF3IP3	5.938131268	5.40557451	6.354653429	0.949079	5.348776

MS4A6A	8.262794199	7.835839929	8.625484051	0.789644	5.152165
IL2RB	7.587283874	7.126831907	7.950180064	0.823348	5.143436
**XCL2**	**5.572395772**	**5.033894474**	**5.983404147**	**0.94951**	**5.023897**
NKG7	5.778787021	5.253638503	6.184705263	0.931067	5.009546
IGHG1	6.587915831	6.093804278	6.960302073	0.866498	4.946329
CD37	6.290297443	5.798995544	6.684224083	0.885229	4.929263
TFEC	5.1728647	4.628177362	5.604133652	0.975956	4.927105
SAMSN1	5.560036578	5.038290909	5.976263955	0.937973	4.891684
GMFG	7.518549066	7.079934938	7.880639394	0.800704	4.82035
SPOCK2	7.207979548	6.761451515	7.576785008	0.815333	4.791639
GIMAP4	7.264250836	6.830286809	7.61834992	0.788063	4.511416
KLRK1	6.889517994	6.438042781	7.245309091	0.807266	4.489753
LPXN	7.746697443	7.32299287	8.083051834	0.760059	4.475187
SASH3	7.170318879	6.737637433	7.521033971	0.783397	4.400497
ARHGAP15	6.990854474	6.553126203	7.339731898	0.786606	4.325581
PTPRCAP	6.707775713	6.254457219	7.046390431	0.791933	4.206837
LCP2	7.113811898	6.692862923	7.459373844	0.766511	4.179642
FYB	6.447662045	6.001651337	6.804338118	0.802687	4.154268
DOCK2	6.134432055	5.682724777	6.498701595	0.815977	4.084416
TNFAIP8	6.963164503	6.533438859	7.296220096	0.762781	4.051414
CCR5	7.945388987	7.55002549	8.261048325	0.711023	4.016819
**WIPF1**	**7.343400688**	**6.934990553**	**7.674235407**	**0.739245**	**4.013043**
RNASE6	7.321141101	6.921656684	7.661933652	0.740277	4.012058
ITK	5.598348869	5.128649733	5.943720415	0.815071	3.719208
PIK3CD	7.138927827	6.747414795	7.459578947	0.712164	3.620706
CD247	6.291976106	5.868597326	6.625066507	0.756469	3.600556
CST7	6.395273746	5.974551515	6.72413748	0.749586	3.593371
SLA	7.718620747	7.345827094	8.017780861	0.671954	3.485126
GPR171	5.250843658	4.784606417	5.593155183	0.808549	3.432745
PRF1	6.402095772	5.999589661	6.707936523	0.708347	3.212285
ARHGAP25	6.674330088	6.288845098	6.978645455	0.6898	3.17581
AIF1	8.848290659	8.528689661	9.120409729	0.59172	3.098075
SEMA4D	7.126236775	6.759564171	7.415976715	0.656413	3.070535
PVRIG	6.425590757	6.060007843	6.709023285	0.649015	2.706594
TNFRSF1B	6.640579056	6.291607843	6.92482823	0.63322	2.66266
GIMAP5	8.061809046	7.742344563	8.312354864	0.57001	2.619376
KLRB1	5.936493805	5.567783244	6.222775279	0.654992	2.546842
NCKAP1L	7.130139921	6.803570588	7.398182616	0.594612	2.520957
INPP5D	7.144708063	6.811960428	7.402840989	0.590881	2.494502
SELPLG	6.99029469	6.664905169	7.255625518	0.59072	2.439267
NCF4	5.325611996	4.956713012	5.624498565	0.667786	2.37489
LST1	7.370344051	7.066526203	7.626305263	0.559779	2.309516
ACAP1	5.93509705	5.581861854	6.202281659	0.62042	2.284542
CD3E	6.862377483	6.542250802	7.117932695	0.575682	2.274258
STAT4	6.167154769	5.826466845	6.422362041	0.595895	2.189902
GPR18	4.753309145	4.367512656	5.036049282	0.668537	2.12445
CD97	6.867657817	6.564857219	7.12053748	0.55568	2.120599

BIN2	6.461629695	6.13912549	6.711647209	0.572522	2.118
IL16	6.383053294	6.060945633	6.625586124	0.56464	2.035038
C3AR1	7.819122616	7.550144742	8.047679266	0.497535	1.93555
PTPN7	6.442389381	6.141385918	6.671142424	0.529757	1.808005
MAP4K1	6.4303588	6.142728877	6.653082456	0.510354	1.674856
LAT	5.945900983	5.644310695	6.172238756	0.527928	1.65717
CTSW	6.470041101	6.19022139	6.680219458	0.489998	1.553445
CD6	7.638147984	7.385813012	7.835034609	0.449222	1.541379
MYO1F	6.988559194	6.730153476	7.19671882	0.466565	1.521292
SP140	4.900231957	4.585762389	5.132966826	0.547204	1.46729
SLAMF1	5.069247886	4.771623529	5.294866986	0.523243	1.387878
AOAH	5.993415241	5.734043137	6.203420893	0.469378	1.320442
PRKCQ	6.170280924	5.910334046	6.372172249	0.461838	1.316087
ITGAL	5.694476106	5.425220677	5.893082775	0.467862	1.246492
FAM65B	4.325567453	4.01714385	4.542195534	0.525052	1.192469
SIRPG	5.298496362	5.032109982	5.495497129	0.463387	1.137734
ZAP70	5.480504326	5.225018717	5.670443381	0.445425	1.087349
TRAT1	4.074480728	3.779438859	4.289792185	0.510353	1.061241
MFNG	6.575046411	6.351592157	6.745508772	0.393917	1.020252
PPP1R16B	4.824646509	4.563967558	5.011176874	0.447209	0.964911
ICOS	4.791651622	4.544464706	4.976512281	0.432048	0.894434
CD7	5.330499902	5.109503922	5.498829346	0.389325	0.807967
CD96	4.462033235	4.219178075	4.642878469	0.4237	0.801033
CRTAM	3.881993805	3.664434046	4.042034769	0.377601	0.553504
UBASH3A	**4.739270403**	**4.550793226**	**4.878039075**	**0.327246**	**0.507528**
**HLA-DRB1**	11.35432242	11.31654813	11.38883349	0.072285	0.059328
NCF1C	5.852842281	5.819620677	5.881574641	0.061954	0.022465
**HLA-DRB6**	7.672151032	7.641781105	7.695711962	0.053931	0.022315
NCF1	5.668487807	5.634323708	5.694335726	0.060012	0.020415

Supplemental Table 3. iRDM 72-gene panel

**Immunity genes(17)**	**Invasion genes(2)**	**Prolif genes(19)**	**Her2 genes (3)**
APOBEC3G	CTSL2	AURKA	HER2
CCL5	MMP11	BIRC5	FGFR4
CCR2	**ER genes (14)**	CCNB1	GRB7
CD2	BAG1	CCNE1	**Basal genes (11)**
CD27	BCL2	CDC20	ACTR3B
CD3D	BLVRA	CDC6	CDH3
CD52	CD68	CENPF	EGFR
CORO1A	ER	CEP55	FOXC1
CXCL9	FOXA1	EXO1	KRT14
GZMA	GSTM1	KIF2C	KRT17
GZMK	MAPT	MELK	KRT5
HLA-DMA	MDM2	Ki67	MIA
IL2RG	MLPH	MYBL2	MYC
LCK	NAT1	NDC80	PHGDH
PRKCB	PGR	ORC6	SFRP1
PTPRC	SCUBE2	PTTG1	TYMS
SH2D1A	SLC39A6	RRM2	UBE2C

**Reference Genes(6)**	GAPDH	GUSB	MRPL19	PSMC4	SF3A1	TFRC

Supplemental Table 4. Composition of subtypes by PAM50 algorithm within iRDM Immuno group in Training and Test data

Immuno Only	PAM50	Basal	HER2E	LumA	LumB	Normal	Mixed	Total
AFFY1951	No.	48	48	51	91	10	94	342
	%	14%	14%	15%	27%	3%	27%	100%
METABRIC	No.	40	33	31	85	26	108	323
	%	12%	10%	10%	26%	8%	33%	100%
TCGA	No.	19	22	35	55	3	37	171
	%	11%	13%	20%	32%	2%	22%	100%
TARGETSEQ	No.	11	24	7	18	2	18	80
	%	14%	30%	9%	22%	2%	22%	100%

Supplemental Table 5. Tils on H&E slides and gene expression Immunity Scores of 91 Triple Negative Breast Cancer

Sample_Name	event_DMFS	DMFS_YEARS	TILs1 percentage	TILs2 percentage	TILs3 percentage	TILs4 percentage	TILs5 percentage	TILsAverge	TIL50_Groups	Immuno_Score	Immuno_Groups	TIL50_ImmunoScore_3Groups	Subtype_by_iRDM	Subtype_by_PAM50
SH_TS_BC111	0	6.67	20	5	5	5	5	8	low	36	istrong	TIL50_low_istrong	Basal	Basal
SH_TS_BC113	0	1.92	30	5	10	5	5	11	low	34	istrong	TIL50_low_istrong	Basal	Basal
SH_TS_BC119	0	6.75	50	40	65	90	40	57	high	45	istrong	TIL50_high_istrong	Her2	Her2
SH_TS_BC133	0	3.92	80	50	60	60	30	56	high	21	iweak	TIL50_high_iweak	Basal	Basal
SH_TS_BC139	1	5.75	20	5	30	5	5	13	low	31	istrong	TIL50_low_istrong	Basal	Basal
SH_TS_BC141	0	8.17	50	25	20	20	15	26	low	36	istrong	TIL50_low_istrong	Basal	Basal
SH_TS_BC146	0	3.42	10	5	5	15	5	8	low	27	iweak	TIL50_low_iweak	Basal	Basal
SH_TS_BC151	0	2.17	80	50	60	90	90	74	high	53	istrong	TIL50_high_istrong	Basal	Basal
SH_TS_BC152	1	0.75	5	15	5	5	5	7	low	1	iweak	TIL50_low_iweak	Basal	Basal
SH_TS_BC153	0	9.25	80	65	50	45	70	62	high	54	istrong	TIL50_high_istrong	Immuno	Her2
SH_TS_BC154	0	0.25	1	5	5	5	1	3.4	low	26	iweak	TIL50_low_iweak	Basal	Basal
SH_TS_BC155	0	9.00	30	5	50	40	20	29	low	39	istrong	TIL50_low_istrong	Basal	Basal
SH_TS_BC160	0	8.42	5	5	5	1	10	5.2	low	38	istrong	TIL50_low_istrong	Basal	Basal
SH_TS_BC163	0	8.00	10	55	15	40	25	29	low	33	istrong	TIL50_low_istrong	Her2	Her2
SH_TS_BC166	0	7.75	50	10	25	10	20	23	low	47	istrong	TIL50_low_istrong	Basal	Basal
SH_TS_BC169	0	7.50	20	10	5	20	25	16	low	38	istrong	TIL50_low_istrong	Her2	Her2
SH_TS_BC170	0	6.83	55	60	30	45	60	50	high	54	istrong	TIL50_high_istrong	Immuno	Her2
SH_TS_BC171	0	7.67	60	70	90	80	80	76	high	80	istrong	TIL50_high_istrong	Immuno	Basal
SH_TS_BC172	0	###	70	30	30	10	50	38	low	20	iweak	TIL50_low_iweak	Basal	Basal
SH_TS_BC173	0	7.50	25	50	15	15	5	22	low	17	iweak	TIL50_low_iweak	Basal	Basal
SH_TS_BC175	0	7.42	30	25	30	40	30	31	low	50	istrong	TIL50_low_istrong	Immuno	Her2
SH_TS_BC176	0	###	50	30	50	30	45	41	low	33	istrong	TIL50_low_istrong	Her2	Her2
SH_TS_BC177	0	7.33	70	50	75	45	50	58	high	47	istrong	TIL50_high_istrong	Her2	Her2
SH_TS_BC180	0	0.50	90	90	80	80	60	80	high	44	istrong	TIL50_high_istrong	Basal	Basal
SH_TS_BC181	0	2.33	70	10	10	5	5	20	low	26	iweak	TIL50_low_iweak	Her2	Her2
SH_TS_BC182	1	1.67	50	35	35	50	45	43	low	57	istrong	TIL50_low_istrong	Immuno	Her2
SH_TS_BC183	0	1.25	5	5	1	15	5	6.2	low	35	istrong	TIL50_low_istrong	Basal	Basal
SH_TS_BC184	0	4.58	50	20	70	90	90	64	high	41	istrong	TIL50_high_istrong	Basal	Basal
SH_TS_BC185	0	2.25	10	5	1	5	10	6.2	low	23	iweak	TIL50_low_iweak	Basal	Basal
SH_TS_BC188	1	1.50	5	15	40	35	10	21	low	49	istrong	TIL50_low_istrong	Basal	Basal
SH_TS_BC193	0	7.00	50	40	10	10	5	23	low	44	istrong	TIL50_low_istrong	Immuno	Basal
SH_TS_BC196	0	0.08	15	10	10	5	40	16	low	37	istrong	TIL50_low_istrong	Her2	Basal
SH_TS_BC200	0	1.58	20	20	20	5	10	15	low	40	istrong	TIL50_low_istrong	Basal	Basal
SH_TS_BC202	0	2.08	60	20	15	25	5	25	low	52	istrong	TIL50_low_istrong	Immuno	Basal

SH_TS_BC203	0	6.92	5	5	5	5	5	5	low	40	istrong	TIL50_low_istrong	Basal	Basal
SH_TS_BC204	0	6.67	10	50	50	20	15	29	low	60	istrong	TIL50_low_istrong	Immuno	Her2
SH_TS_BC207	0	5.50	70	80	60	90	60	72	high	35	istrong	TIL50_high_istrong	Basal	Basal
SH_TS_BC211	1	0.33	5	5	5	10	5	6	low	53	istrong	TIL50_low_istrong	Immuno	Her2
SH_TS_BC212	0	6.42	5	5	10	5	5	6	low	34	istrong	TIL50_low_istrong	Her2	Her2
SH_TS_BC213	0	7.75	5	5	5	10	5	6	low	31	istrong	TIL50_low_istrong	Basal	Basal
SH_TS_BC214	0	0.83	70	60	70	60	40	60	high	28	iweak	TIL50_high_iweak	Basal	Basal
SH_TS_BC220	1	0.83	5	5	5	1	10	5.2	low	19	iweak	TIL50_low_iweak	Basal	Basal
SH_TS_BC222	1	4.08	40	45	30	35	30	36	low	22	iweak	TIL50_low_iweak	Basal	Basal
SH_TS_BC226	0	5.17	5	5	5	1	1	3.4	low	39	istrong	TIL50_low_istrong	Basal	Basal
SH_TS_BC232	1	1.00	10	5	5	5	5	6	low	28	iweak	TIL50_low_iweak	Her2	Basal
SH_TS_BC236	1	2.83	35	5	10	15	50	23	low	41	istrong	TIL50_low_istrong	Basal	Basal
SH_TS_BC237	1	2.17	40	60	20	20	90	46	low	28	iweak	TIL50_low_iweak	Basal	Basal
SH_TS_BC239	0	1.42	5	5	5	5	5	5	low	31	istrong	TIL50_low_istrong	Basal	Basal
SH_TS_BC240	0	2.67	90	80	70	75	70	77	high	71	istrong	TIL50_high_istrong	Immuno	Basal
SH_TS_BC243	0	4.00	30	20	50	60	50	42	low	37	istrong	TIL50_low_istrong	Basal	Basal
SH_TS_BC246	0	4.92	60	10	15	10	35	26	low	21	iweak	TIL50_low_iweak	Her2	Her2
SH_TS_BC247	1	1.33	20	5	30	5	5	13	low	34	istrong	TIL50_low_istrong	Basal	Basal
SH_TS_BC249	0	5.25	80	25	75	85	60	65	high	52	istrong	TIL50_high_istrong	Basal	Basal
SH_TS_BC251	0	2.75	5	15	10	20	5	11	low	40	istrong	TIL50_low_istrong	Basal	Basal
SH_TS_BC252	0	5.25	40	60	50	40	60	50	high	56	istrong	TIL50_high_istrong	LumA	Basal
SH_TS_BC253	0	5.25	10	5	1	10	5	6.2	low	31	istrong	TIL50_low_istrong	Basal	Basal
SH_TS_BC255	0	5.08	10	40	40	35	20	29	low	72	istrong	TIL50_low_istrong	Immuno	Her2
SH_TS_BC257	0	5.50	80	70	80	80	50	72	high	41	istrong	TIL50_high_istrong	Basal	Basal
SH_TS_BC258	1	1.67	20	20	25	55	20	28	low	41	istrong	TIL50_low_istrong	Basal	Basal
SH_TS_BC260	0	5.50	10	5	10	35	40	20	low	32	istrong	TIL50_low_istrong	Basal	Basal
SH_TS_BC262	0	5.58	40	30	20	20	40	30	low	11	iweak	TIL50_low_iweak	Basal	Basal
SH_TS_BC264	1	1.42	15	15	10	15	5	12	low	32	istrong	TIL50_low_istrong	Basal	Basal
SH_TS_BC265	0	5.33	65	80	25	30	50	50	high	42	istrong	TIL50_high_istrong	Basal	Basal
SH_TS_BC266	0	3.58	20	15	5	10	25	15	low	43	istrong	TIL50_low_istrong	Basal	Basal
SH_TS_BC271	0	4.33	10	20	30	20	15	19	low	37	istrong	TIL50_low_istrong	LumA	Basal
SH_TS_BC272	0	4.42	80	90	70	80	80	80	high	55	istrong	TIL50_high_istrong	Basal	Basal
SH_TS_BC273	0	4.58	10	5	5	5	5	6	low	36	istrong	TIL50_low_istrong	Basal	Her2
SH_TS_BC281	0	4.00	60	30	50	40	25	41	low	38	istrong	TIL50_low_istrong	Her2	Her2
SH_TS_BC283	0	###	40	30	30	20	35	31	low	43	istrong	TIL50_low_istrong	Her2	Her2
SH_TS_BC284	0	5.00	50	40	35	10	45	36	low	47	istrong	TIL50_low_istrong	LumA	Her2
SH_TS_BC285	0	5.92	15	10	2	20	15	12	low	27	iweak	TIL50_low_iweak	Basal	Basal
SH_TS_BC286	0	6.00	5	2	10	5	15	7.4	low	45	istrong	TIL50_low_istrong	Basal	Basal
SH_TS_BC287	1	0.04	5	1	1	2	2	2.2	low	27	iweak	TIL50_low_iweak	Basal	Basal
SH_TS_BC290	0	8.92	10	15	80	10	20	27	low	47	istrong	TIL50_low_istrong	Basal	Basal
SH_TS_BC296	0	5.83	50	80	60	50	60	60	high	44	istrong	TIL50_high_istrong	Basal	Basal
SH_TS_BC1805004	1	2.67	10	15	5	5	10	9	low	34	istrong	TIL50_low_istrong	Basal	Basal
SH_TS_BC1805005	0	7.83	25	25	5	5	10	14	low	34	istrong	TIL50_low_istrong	Her2	Her2
SH_TS_BC1805006	1	1.92	10	5	5	15	20	11	low	39	istrong	TIL50_low_istrong	Basal	Basal
SH_TS_BC1805008	1	0.92	10	25	15	10	5	13	low	46	istrong	TIL50_low_istrong	Basal	Basal
SH_TS_BC1805009	0	7.67	50	30	35	60	50	45	low	69	istrong	TIL50_low_istrong	LumA	Basal
SH_TS_BC1805010	0	4.83	80	60	90	50	80	72	high	70	istrong	TIL50_high_istrong	Basal	Basal

SH_TS_BC1805011	0	4.92	80	60	70	40	50	60	high	65	istrong	TIL50_high_istrong	Basal	Basal
SH_TS_BC1805013	0	5.08	20	10	10	10	10	12	low	42	istrong	TIL50_low_istrong	Basal	Basal
SH_TS_BC1805015	1	1.42	30	5	5	5	20	13	low	36	istrong	TIL50_low_istrong	Her2	Her2
SH_TS_BC1805016	0	4.92	25	50	70	55	40	48	low	44	istrong	TIL50_low_istrong	Basal	Basal
SH_TS_BC1805017	0	5.92	10	15	25	20	15	17	low	54	istrong	TIL50_low_istrong	Basal	Basal
SH_TS_BC1805018	1	0.17	30	10	5	10	5	12	low	27	iweak	TIL50_low_iweak	Basal	Her2
SH_TS_BC1805019	1	0.58	25	5	10	15	10	13	low	53	istrong	TIL50_low_istrong	Basal	Basal
SH_TS_BC307	0	5.58	50	50	15	25	70	42	low	45	istrong	TIL50_low_istrong	Her2	Her2
SH_TS_BC310	0	2.08	80	90	15	30	20	47	low	64	istrong	TIL50_low_istrong	Immuno	Her2
SH_TS_BC376	1	1.58	35	30	20	15	10	22	low	18	iweak	TIL50_low_iweak	Her2	Her2

Supplemental Table 6. DMFS time and event of 303 pateitients in TAREGETSEQ dataset.

TARGETSEQ_Sample	DMFS_TIME(YEARS)	DMFS_EVENT	ER_status	iRDM_subtypes	Proliferation_Groups	Immunity_Groups	iRDM_score	iRDM_risk_group
SH_TS_BC111	6.67	0	ER+	Basal	high	iweak	57	high
SH_TS_BC112	6.08	0	ER+	LumA	low	iweak	16	low
SH_TS_BC113	1.92	0	ER-	Basal	high	iweak	57	high
SH_TS_BC119	6.75	0	ER-	Her2	high	istrong	48	med
SH_TS_BC133	3.92	0	ER+	Basal	high	iweak	93	high
SH_TS_BC134	11.00	0	ER+	LumA	low	iweak	40	med
SH_TS_BC139	5.75	1	ER+	Basal	high	iweak	75	high
SH_TS_BC141	8.17	0	ER+	Basal	low	iweak	63	high
SH_TS_BC146	3.42	0	ER+	Basal	high	iweak	85	high
SH_TS_BC147	9.75	0	ER+	LumB	high	iweak	57	high
SH_TS_BC150	9.08	0	ER+	LumA	low	istrong	1	low
SH_TS_BC151	2.17	0	ER-	Basal	high	istrong	38	med
SH_TS_BC152	0.75	1	ER+	Basal	high	iweak	81	high
SH_TS_BC153	9.25	0	ER+	Immuno	high	istrong	61	high
SH_TS_BC154	0.25	0	ER-	Basal	high	iweak	66	high
SH_TS_BC155	9.00	0	ER+	Basal	high	iweak	61	high
SH_TS_BC160	8.42	0	ER-	Basal	high	iweak	59	high
SH_TS_BC161	3.17	0	ER-	Her2	high	iweak	59	high
SH_TS_BC163	8.00	0	ER-	Her2	high	iweak	60	high
SH_TS_BC165	7.75	0	ER+	Immuno	low	istrong	26	low
SH_TS_BC166	7.75	0	ER-	Basal	high	istrong	50	high
SH_TS_BC169	7.50	0	ER+	Her2	high	iweak	63	high
SH_TS_BC170	6.83	0	ER+	Immuno	low	istrong	41	med
SH_TS_BC171	7.67	0	ER+	Immuno	low	istrong	33	med
SH_TS_BC172	13.17	0	ER-	Basal	high	iweak	60	high
SH_TS_BC173	7.50	0	ER-	Basal	high	iweak	61	high
SH_TS_BC175	7.42	0	ER-	Immuno	low	istrong	49	med
SH_TS_BC176	10.33	0	ER+	Her2	high	iweak	68	high
SH_TS_BC177	7.33	0	ER-	Her2	high	istrong	47	med
SH_TS_BC178	7.00	0	ER+	LumA	low	istrong	4	low
SH_TS_BC180	0.50	0	ER+	Basal	high	istrong	68	high
SH_TS_BC181	2.33	0	ER-	Her2	high	iweak	63	high
SH_TS_BC182	1.67	1	ER-	Immuno	low	istrong	40	med
SH_TS_BC183	1.25	0	ER-	Basal	high	iweak	63	high
SH_TS_BC184	4.58	0	ER+	Basal	high	iweak	76	high
SH_TS_BC185	2.25	0	ER-	Basal	high	iweak	64	high
SH_TS_BC188	1.50	1	ER+	Basal	high	istrong	78	high
SH_TS_BC193	7.00	0	ER-	Immuno	high	istrong	37	med
SH_TS_BC196	0.08	0	ER+	Her2	high	iweak	82	high

SH_TS_BC198	6.75	0	ER+	LumB	high	iweak	57	high
SH_TS_BC199	7.00	0	ER+	LumA	low	istrong	24	low
SH_TS_BC200	1.58	0	ER+	Basal	high	iweak	66	high
SH_TS_BC202	2.08	0	ER+	Immuno	low	istrong	45	med
SH_TS_BC203	6.92	0	ER+	Basal	high	iweak	53	high
SH_TS_BC204	6.67	0	ER-	Immuno	high	istrong	35	med
SH_TS_BC207	5.50	0	ER+	Basal	high	iweak	88	high
SH_TS_BC209	0.42	1	ER+	Basal	high	istrong	75	high
SH_TS_BC210	2.25	0	ER+	LumA	low	iweak	15	low
SH_TS_BC211	0.33	1	ER-	Immuno	high	istrong	36	med
SH_TS_BC212	6.42	0	ER-	Her2	high	iweak	59	high
SH_TS_BC213	7.75	0	ER+	Basal	high	iweak	80	high
SH_TS_BC214	0.83	0	ER-	Basal	high	iweak	59	high
SH_TS_BC219	5.08	0	ER+	LumA	low	istrong	32	low
SH_TS_BC220	0.83	1	ER-	Basal	high	iweak	65	high
SH_TS_BC222	4.08	1	ER-	Basal	high	iweak	64	high
SH_TS_BC226	5.17	0	ER+	Basal	low	iweak	63	high
SH_TS_BC232	1.00	1	ER-	Her2	high	iweak	62	high
SH_TS_BC233	1.92	0	ER+	LumA	low	istrong	17	low
SH_TS_BC235	2.08	0	ER+	LumA	low	istrong	20	low
SH_TS_BC236	2.83	1	ER+	Basal	high	iweak	67	high
SH_TS_BC237	2.17	1	ER+	Basal	high	iweak	65	high
SH_TS_BC239	1.42	0	ER-	Basal	high	iweak	63	high
SH_TS_BC240	2.67	0	ER-	Immuno	low	istrong	58	high
SH_TS_BC243	4.00	0	ER-	Basal	high	iweak	60	high
SH_TS_BC246	4.92	0	ER+	Her2	high	iweak	87	high
SH_TS_BC247	1.33	1	ER-	Basal	high	iweak	59	high
SH_TS_BC249	5.25	0	ER+	Basal	low	istrong	55	high
SH_TS_BC251	2.75	0	ER-	Basal	high	iweak	49	med
SH_TS_BC252	5.25	0	ER-	LumA	low	istrong	50	high
SH_TS_BC253	5.25	0	ER+	Basal	high	iweak	70	high
SH_TS_BC255	5.08	0	ER-	Immuno	low	istrong	45	med
SH_TS_BC257	5.50	0	ER+	Basal	high	iweak	74	high
SH_TS_BC258	1.67	1	ER+	Basal	low	iweak	70	high
SH_TS_BC260	5.50	0	ER-	Basal	high	iweak	57	high
SH_TS_BC262	5.58	0	ER-	Basal	high	iweak	61	high
SH_TS_BC264	1.42	1	ER-	Basal	high	iweak	55	high
SH_TS_BC265	5.33	0	ER+	Basal	low	istrong	63	high
SH_TS_BC266	3.58	0	ER-	Basal	high	istrong	49	med
SH_TS_BC271	4.33	0	ER+	LumA	low	iweak	52	high
SH_TS_BC272	4.42	0	ER-	Basal	high	istrong	38	med
SH_TS_BC273	4.58	0	ER+	Basal	high	iweak	73	high
SH_TS_BC281	4.00	0	ER-	Her2	high	iweak	58	high
SH_TS_BC283	17.00	0	ER+	Her2	high	istrong	71	high
SH_TS_BC284	5.00	0	ER+	LumA	low	istrong	51	high
SH_TS_BC285	5.92	0	ER+	Basal	high	iweak	58	high
SH_TS_BC286	6.00	0	ER+	Basal	high	istrong	69	high

SH_TS_BC287	0.04	1	ER+	Basal	high	iweak	78	high
SH_TS_BC289	3.42	1	ER+	LumA	low	istrong	23	low
SH_TS_BC290	8.92	0	ER+	Basal	high	istrong	80	high
SH_TS_BC291	6.00	0	ER+	LumA	low	istrong	45	med
SH_TS_BC296	5.83	0	ER-	Basal	high	istrong	48	med
UNC_TS_BC01	5.17	0	ER+	Immuno	low	istrong	31	low
UNC_TS_BC02	5.17	0	ER+	LumA	low	iweak	22	low
UNC_TS_BC03	5.08	0	ER+	LumA	low	iweak	29	low
UNC_TS_BC04	5.58	0	ER+	LumB	high	iweak	47	med
UNC_TS_BC06	1.08	0	ER+	Immuno	high	istrong	42	med
UNC_TS_BC07	4.33	0	ER+	Immuno	high	istrong	63	high
UNC_TS_BC10	4.92	0	ER+	Basal	high	iweak	83	high
UNC_TS_BC105	2.75	0	ER-	LumA	low	istrong	49	med
UNC_TS_BC106	6.00	0	ER-	Immuno	low	istrong	53	high
UNC_TS_BC109	5.83	0	ER+	LumA	low	istrong	12	low
UNC_TS_BC11	5.50	0	ER-	Immuno	high	istrong	39	med
UNC_TS_BC110	5.75	0	ER+	Basal	low	iweak	58	high
UNC_TS_BC111	5.58	0	ER+	LumA	low	istrong	19	low
UNC_TS_BC113	5.75	0	ER+	Immuno	low	istrong	21	low
UNC_TS_BC115	5.92	0	ER+	Immuno	high	istrong	48	med
UNC_TS_BC118	5.25	0	ER+	LumB	high	iweak	54	high
UNC_TS_BC119	4.33	0	ER+	LumB	high	istrong	46	med
UNC_TS_BC120	1.75	0	ER+	LumA	low	iweak	17	low
UNC_TS_BC121	3.92	0	ER+	LumB	high	istrong	40	med
UNC_TS_BC122	5.58	0	ER+	Immuno	high	istrong	51	high
UNC_TS_BC123	5.83	0	ER+	LumA	low	iweak	26	low
UNC_TS_BC124	6.08	0	ER+	LumB	high	iweak	42	med
UNC_TS_BC128	6.08	0	ER+	Immuno	high	istrong	42	med
UNC_TS_BC129	6.42	0	ER+	LumA	low	iweak	28	low
UNC_TS_BC130	5.08	0	ER+	Immuno	low	istrong	34	med
UNC_TS_BC131	3.08	0	ER+	LumB	low	istrong	30	low
UNC_TS_BC132	6.50	0	ER+	LumB	high	istrong	52	high
UNC_TS_BC133	0.50	1	ER-	Basal	high	istrong	50	high
UNC_TS_BC134	6.25	0	ER+	LumB	high	istrong	14	low
UNC_TS_BC135	5.42	0	ER+	LumA	low	istrong	17	low
UNC_TS_BC136	6.67	0	ER+	LumA	low	istrong	14	low
UNC_TS_BC137	5.67	0	ER+	LumA	low	iweak	59	high
UNC_TS_BC138	5.42	0	ER+	LumA	low	iweak	8	low
UNC_TS_BC139	6.33	0	ER+	Immuno	low	istrong	26	low
UNC_TS_BC140	5.92	0	ER-	Basal	high	istrong	47	med
UNC_TS_BC141	0.92	0	ER+	LumA	low	iweak	14	low
UNC_TS_BC142	5.25	0	ER+	LumA	low	istrong	22	low
UNC_TS_BC143	4.17	0	ER+	Her2	high	istrong	67	high
UNC_TS_BC144	6.08	0	ER-	Her2	high	istrong	51	high
UNC_TS_BC145	5.58	0	ER+	LumA	low	istrong	3	low
UNC_TS_BC146	4.75	0	ER+	LumA	low	iweak	38	med
UNC_TS_BC148	1.75	0	ER-	Basal	high	iweak	58	high

UNC_TS_BC149	6.50	0	ER+	LumA	low	iweak	29	low
UNC_TS_BC150	4.83	0	ER+	LumA	low	istrong	5	low
UNC_TS_BC151	5.33	0	ER+	Her2	high	istrong	75	high
UNC_TS_BC152	0.00	1	ER-	Immuno	low	istrong	49	med
UNC_TS_BC153	2.00	0	ER+	Immuno	low	istrong	18	low
UNC_TS_BC154	6.08	0	ER+	LumA	low	istrong	17	low
UNC_TS_BC155	5.58	0	ER+	LumA	low	iweak	26	low
UNC_TS_BC156	5.08	0	ER-	Immuno	high	istrong	37	med
UNC_TS_BC157	6.83	0	ER+	LumA	low	istrong	23	low
UNC_TS_BC158	4.83	1	ER-	Basal	high	iweak	61	high
UNC_TS_BC159	1.17	0	ER+	LumA	low	istrong	13	low
UNC_TS_BC16	0.50	0	ER+	LumB	high	istrong	45	med
UNC_TS_BC160	5.92	0	ER+	LumB	high	iweak	29	low
UNC_TS_BC161	6.25	0	ER+	LumB	high	iweak	50	high
UNC_TS_BC162	5.92	0	ER+	LumA	low	istrong	4	low
UNC_TS_BC163	6.75	0	ER+	LumB	high	iweak	47	med
UNC_TS_BC164	5.58	0	ER+	LumB	high	iweak	42	med
UNC_TS_BC165	6.00	0	ER+	LumB	low	iweak	32	low
UNC_TS_BC166	6.67	0	ER+	Her2	high	iweak	90	high
UNC_TS_BC167	5.67	0	ER+	Immuno	low	istrong	46	med
UNC_TS_BC168	6.42	0	ER+	LumB	high	istrong	54	high
UNC_TS_BC169	6.58	0	ER-	Immuno	low	istrong	42	med
UNC_TS_BC17	4.75	0	ER+	LumA	low	istrong	29	low
UNC_TS_BC170	3.67	0	ER+	Basal	high	istrong	67	high
UNC_TS_BC171	6.42	0	ER-	LumA	low	istrong	28	low
UNC_TS_BC172	6.42	0	ER+	LumA	low	istrong	22	low
UNC_TS_BC173	7.33	0	ER+	Her2	low	istrong	49	med
UNC_TS_BC174	5.50	0	ER-	Basal	high	iweak	55	high
UNC_TS_BC175	6.92	0	ER+	LumA	high	iweak	29	low
UNC_TS_BC176	2.75	0	ER+	LumB	high	istrong	56	high
UNC_TS_BC177	3.08	1	ER+	LumB	high	iweak	68	high
UNC_TS_BC178	7.33	0	ER+	Immuno	low	iweak	21	low
UNC_TS_BC179	4.42	1	ER+	Her2	high	istrong	69	high
UNC_TS_BC180	7.00	0	ER+	Basal	high	iweak	81	high
UNC_TS_BC181	7.17	0	ER+	Immuno	high	istrong	56	high
UNC_TS_BC182	7.08	0	ER+	Her2	high	iweak	59	high
UNC_TS_BC184	7.17	0	ER+	Immuno	low	istrong	54	high
UNC_TS_BC185	6.33	0	ER+	LumA	low	iweak	32	low
UNC_TS_BC187	7.08	0	ER+	Basal	high	iweak	84	high
UNC_TS_BC188	7.17	0	ER+	Immuno	low	istrong	21	low
UNC_TS_BC189	7.08	0	ER+	LumA	low	iweak	27	low
UNC_TS_BC19	3.50	0	ER+	Her2	high	istrong	66	high
UNC_TS_BC190	5.92	0	ER+	LumA	low	istrong	28	low
UNC_TS_BC193	6.92	0	ER+	LumA	low	iweak	41	med
UNC_TS_BC194	0.58	1	ER-	Basal	high	iweak	58	high
UNC_TS_BC195	7.00	0	ER+	Immuno	low	istrong	34	med
UNC_TS_BC196	3.08	0	ER+	LumA	low	istrong	12	low

UNC_TS_BC198	6.83	1	ER+	Her2	high	iweak	56	high
UNC_TS_BC202	7.75	0	ER+	Basal	high	istrong	92	high
UNC_TS_BC203	6.00	0	ER+	Immuno	low	istrong	33	med
UNC_TS_BC205	7.25	0	ER+	LumA	low	istrong	12	low
UNC_TS_BC206	7.58	0	ER-	LumA	low	istrong	35	med
UNC_TS_BC208	7.17	0	ER-	Her2	high	istrong	48	med
UNC_TS_BC210	6.67	0	ER+	LumA	low	iweak	29	low
UNC_TS_BC212	7.08	0	ER-	Basal	high	istrong	43	med
UNC_TS_BC213	6.75	0	ER+	LumB	high	iweak	44	med
UNC_TS_BC214	3.17	0	ER+	LumA	low	istrong	20	low
UNC_TS_BC216	7.00	0	ER+	Immuno	low	istrong	32	low
UNC_TS_BC218	7.42	0	ER+	LumA	low	istrong	1	low
UNC_TS_BC219	6.25	0	ER+	LumA	low	iweak	19	low
UNC_TS_BC220	5.42	0	ER+	Immuno	low	istrong	37	med
UNC_TS_BC221	7.25	0	ER+	LumA	low	istrong	23	low
UNC_TS_BC222	5.00	1	ER+	Her2	high	iweak	79	high
UNC_TS_BC223	4.83	0	ER+	LumA	high	iweak	23	low
UNC_TS_BC224	5.83	0	ER-	Basal	high	iweak	56	high
UNC_TS_BC227	7.58	0	ER+	Immuno	low	istrong	32	low
UNC_TS_BC228	7.00	0	ER+	LumA	low	istrong	14	low
UNC_TS_BC231	7.50	0	ER+	Her2	high	iweak	75	high
UNC_TS_BC232	2.75	1	ER+	Immuno	low	istrong	28	low
UNC_TS_BC234	7.83	0	ER+	Immuno	high	istrong	51	high
UNC_TS_BC235	8.00	0	ER+	Basal	low	iweak	46	med
UNC_TS_BC237	8.08	0	ER+	LumB	high	istrong	48	med
UNC_TS_BC238	8.00	0	ER-	Basal	low	istrong	61	high
UNC_TS_BC241	2.33	0	ER-	Basal	high	iweak	53	high
UNC_TS_BC242	7.50	0	ER+	LumA	low	iweak	16	low
UNC_TS_BC243	8.17	0	ER+	LumA	low	istrong	13	low
UNC_TS_BC244	0.08	1	ER-	Her2	low	istrong	63	high
UNC_TS_BC245	7.75	0	ER+	LumA	low	istrong	22	low
UNC_TS_BC246	3.08	1	ER-	Basal	low	istrong	67	high
UNC_TS_BC248	1.67	1	ER+	Basal	low	iweak	57	high
UNC_TS_BC249	8.25	0	ER+	Immuno	low	istrong	16	low
UNC_TS_BC250	0.17	0	ER+	LumB	high	iweak	42	med
UNC_TS_BC251	1.58	0	ER+	LumA	low	iweak	20	low
UNC_TS_BC252	8.08	0	ER+	LumA	low	iweak	32	low
UNC_TS_BC254	8.17	0	ER+	Basal	high	iweak	90	high
UNC_TS_BC255	1.50	0	ER-	Her2	high	istrong	50	high
UNC_TS_BC257	7.83	0	ER+	LumA	low	iweak	45	med
UNC_TS_BC258	7.08	0	ER+	Basal	high	istrong	78	high
UNC_TS_BC259	8.08	0	ER+	LumA	low	istrong	27	low
UNC_TS_BC26	4.67	0	ER+	LumB	high	istrong	25	low
UNC_TS_BC265	2.42	0	ER-	Her2	high	iweak	63	high
UNC_TS_BC267	1.75	0	ER+	LumA	low	istrong	10	low
UNC_TS_BC268	4.92	0	ER+	LumB	high	iweak	36	med
UNC_TS_BC269	7.83	0	ER+	LumA	low	iweak	13	low

UNC_TS_BC27	4.42	0	ER+	LumA	low	iweak	30	low
UNC_TS_BC270	4.58	1	ER+	LumB	high	iweak	44	med
UNC_TS_BC271	0.83	0	ER-	Basal	high	istrong	58	high
UNC_TS_BC272	5.00	0	ER-	Basal	high	iweak	62	high
UNC_TS_BC273	7.67	0	ER+	LumA	low	istrong	10	low
UNC_TS_BC274	8.67	0	ER+	LumA	low	istrong	25	low
UNC_TS_BC275	6.33	0	ER+	LumA	low	istrong	19	low
UNC_TS_BC276	7.92	0	ER+	LumB	high	istrong	29	low
UNC_TS_BC277	6.75	0	ER+	LumA	low	iweak	44	med
UNC_TS_BC278	8.42	0	ER+	LumA	low	istrong	2	low
UNC_TS_BC279	7.75	0	ER-	Immuno	high	istrong	32	low
UNC_TS_BC28	0.17	0	ER+	LumA	low	istrong	54	high
UNC_TS_BC283	8.50	0	ER-	Basal	high	istrong	52	high
UNC_TS_BC284	2.92	1	ER+	Basal	high	istrong	88	high
UNC_TS_BC285	8.17	0	ER-	Basal	high	istrong	52	high
UNC_TS_BC287	7.00	0	ER+	Immuno	high	istrong	56	high
UNC_TS_BC289	5.25	0	ER+	Immuno	low	istrong	37	med
UNC_TS_BC29	4.33	0	ER+	LumA	high	iweak	43	med
UNC_TS_BC290	8.00	0	ER-	LumA	low	istrong	30	low
UNC_TS_BC292	6.67	0	ER+	LumA	low	istrong	23	low
UNC_TS_BC293	13.58	0	ER+	LumA	low	istrong	9	low
UNC_TS_BC294	14.58	0	ER+	LumB	high	iweak	71	high
UNC_TS_BC295	12.58	0	ER+	LumA	low	iweak	31	low
UNC_TS_BC296	8.42	0	ER+	Basal	high	istrong	76	high
UNC_TS_BC30	1.33	0	ER+	LumA	low	istrong	31	low
UNC_TS_BC31	4.50	0	ER+	LumB	high	iweak	60	high
UNC_TS_BC32	5.00	0	ER+	Immuno	high	istrong	37	med
UNC_TS_BC33	1.83	0	ER+	Her2	high	istrong	79	high
UNC_TS_BC34	0.50	0	ER+	Her2	high	istrong	72	high
UNC_TS_BC36	4.50	0	ER-	Basal	high	iweak	66	high
UNC_TS_BC37	4.33	0	ER+	Immuno	low	istrong	34	med
UNC_TS_BC38	4.92	0	ER-	LumA	low	iweak	40	med
UNC_TS_BC41	4.17	1	ER-	Immuno	low	istrong	39	med
UNC_TS_BC42	4.08	0	ER+	LumB	high	iweak	47	med
UNC_TS_BC45	5.00	0	ER-	Immuno	low	istrong	59	high
UNC_TS_BC46	5.08	0	ER+	LumB	high	iweak	41	med
UNC_TS_BC47	4.83	0	ER+	LumB	high	iweak	56	high
UNC_TS_BC49	1.50	1	ER+	LumA	low	istrong	50	high
UNC_TS_BC52	5.50	0	ER+	LumA	high	iweak	21	low
UNC_TS_BC53	5.58	0	ER+	LumA	low	iweak	23	low
UNC_TS_BC54	5.25	0	ER+	LumA	low	istrong	28	low
UNC_TS_BC55	5.17	0	ER+	LumA	low	istrong	15	low
UNC_TS_BC56	5.17	0	ER+	LumA	low	iweak	42	med
UNC_TS_BC57	5.67	0	ER+	Basal	low	iweak	54	high
UNC_TS_BC58	4.17	0	ER+	LumA	low	iweak	32	low
UNC_TS_BC59	4.75	0	ER+	Immuno	high	istrong	64	high
UNC_TS_BC60	5.08	0	ER+	LumA	low	istrong	21	low

UNC_TS_BC61	5.50	0	ER-	Her2	high	istrong	43	med
UNC_TS_BC62	0.67	0	ER+	LumB	high	iweak	43	med
UNC_TS_BC63	5.58	0	ER+	LumA	low	iweak	22	low
UNC_TS_BC66	3.50	1	ER+	LumA	low	istrong	35	med
UNC_TS_BC67	5.33	0	ER+	LumA	low	iweak	18	low
UNC_TS_BC70	0.83	0	ER+	LumA	low	iweak	36	med
UNC_TS_BC72	5.33	0	ER+	LumA	low	iweak	8	low
UNC_TS_BC73	5.58	0	ER-	Basal	high	istrong	48	med
UNC_TS_BC74	5.58	0	ER+	LumB	high	iweak	44	med
UNC_TS_BC75	1.25	1	ER-	Basal	high	iweak	57	high
UNC_TS_BC76	5.42	0	ER+	LumA	low	istrong	36	med
UNC_TS_BC77	1.08	1	ER-	Basal	high	iweak	61	high
UNC_TS_BC78	4.50	0	ER+	LumA	low	iweak	21	low
UNC_TS_BC79	5.17	0	ER-	Basal	high	iweak	55	high
UNC_TS_BC81	2.50	0	ER+	LumB	high	iweak	39	med
UNC_TS_BC82	2.00	0	ER-	Basal	high	iweak	48	med
UNC_TS_BC84	1.50	0	ER-	Basal	high	istrong	36	med
UNC_TS_BC85	3.58	0	ER+	LumA	low	iweak	29	low
UNC_TS_BC86	5.92	0	ER+	LumA	low	iweak	15	low
UNC_TS_BC87	1.00	0	ER+	LumA	low	iweak	15	low
UNC_TS_BC88	5.58	0	ER+	LumA	low	iweak	50	high
UNC_TS_BC90	0.08	1	ER+	LumB	high	iweak	46	med
UNC_TS_BC91	5.75	0	ER+	LumB	high	iweak	54	high
UNC_TS_BC92	5.58	0	ER-	Basal	high	istrong	41	med
UNC_TS_BC93	5.33	0	ER+	LumA	low	iweak	10	low
UNC_TS_BC94	4.83	0	ER+	LumA	low	iweak	10	low
UNC_TS_BC95	5.42	0	ER-	Basal	high	iweak	59	high
UNC_TS_BC97	13.75	0	ER-	Basal	high	istrong	51	high
UNC_TS_BC98	0.00	1	ER-	Her2	high	iweak	57	high
